# Forebrain Shh overexpression improves cognitive function and locomotor hyperactivity in an aneuploid mouse model of Down syndrome and its euploid littermates

**DOI:** 10.1186/s40478-021-01237-z

**Published:** 2021-08-16

**Authors:** Feng J. Gao, Donna Klinedinst, Fabian-Xosé Fernandez, Bei Cheng, Alena Savonenko, Benjamin Devenney, Yicong Li, Dan Wu, Martin G. Pomper, Roger H. Reeves

**Affiliations:** 1 Department of Physiology, Johns Hopkins University, Baltimore, MD 21205 USA; 2 Department of Genetic Medicine, John Hopkins University, Baltimore, MD 21205 USA; 3 Department of Psychology, University of Arizona, Tucson, AZ USA; 4 Department of Neurology, University of Arizona, Tucson, AZ USA; 5 BIO5 and McKnight Brain Research Institutes, Tucson, AZ USA; 6 Department of Radiology, Johns Hopkins University, Baltimore, MD 21205 USA; 7 Department of Pathology and Neurology, John Hopkins University, Baltimore, MD 21205 USA; 8 Department of Biomedical Engineering, Zhejiang University, Hangzhou, 310058 Zhejiang China

**Keywords:** Down Syndrome, Sonic hedgehog, Cognitive function, Hyperactivity, Ts65Dn, TRE-hShh

## Abstract

Down syndrome (DS) is the leading genetic cause of intellectual disability and causes early-onset dementia and cerebellar hypoplasia. The prevalence of attention deficit hyperactivity disorder is elevated in children with DS. The aneuploid DS mouse model “Ts65Dn” shows prominent brain phenotypes, including learning and memory deficits, cerebellar hypoplasia, and locomotor hyperactivity. Previous studies indicate that impaired Sonic hedgehog (Shh) signaling contributes to neurological phenotypes associated with DS and neurodegenerative diseases. However, because of a lack of working inducible Shh knock-in mice, brain region-specific Shh overexpression and its effects on cognitive function have not been studied in vivo. Here, with Gli1-LacZ reporter mice, we demonstrated that Ts65Dn had reduced levels of Gli1, a sensitive readout of Shh signaling, in both hippocampus and cerebellum at postnatal day 6. Through site-specific transgenesis, we generated an inducible human Shh knock-in mouse, TRE-bi-hShh-Zsgreen1 (TRE-hShh), simultaneously expressing dually-lipidated Shh-Np and Zsgreen1 marker in the presence of transactivator (tTA). Double transgenic mice “Camk2a-tTA;TRE-hShh” and “Pcp2-tTA;TRE-hShh” induced Shh overexpression and activated Shh signaling in a forebrain and cerebellum, respectively, specific manner from the perinatal period. Camk2a-tTA;TRE-hShh normalized locomotor hyperactivity and improved learning and memory in 3-month-old Ts65Dn, mitigated early-onset severe cognitive impairment in 7-month-old Ts65Dn, and enhanced spatial cognition in euploid mice. Camk2a-tTA;TRE-hShh cohort maintained until 600days old showed that chronic overexpression of Shh in forebrain from the perinatal period had no effect on longevity of euploid or Ts65Dn. Pcp2-tTA;TRE-hShh did not affect cognition but mitigated the phenotype of cerebellar hypoplasia in Ts65Dn. Our study provides the first in vivo evidence that Shh overexpression from the perinatal period protects DS brain integrity and enhances learning and memory in normal mice, indicating the broad therapeutic potential of Shh ligand for other neurological conditions. Moreover, the first inducible hShh site-specific knock-in mouse could be widely used for spatiotemporal Shh signaling regulation.

**Supplementary Information:**

The online version contains supplementary material available at 10.1186/s40478-021-01237-z.

## Introduction

Sonic hedgehog (Shh), a secreted signaling protein, is essential for morphogenesis and neurogenesis in the developing brain and the maintenance of adult neurogenic niches [59, 84]. During gastrulation, both the prechordal plate and the notochord produce and form Shh gradients to regulate the morphology of the central nervous system [3]. From mouse embryonic day (E) 8 to E10.5, Shh from the notochord induces primary neurulation to form the neural tube [37, 60, 85]. The anterior neural tube develops into forebrain, midbrain, and hindbrain (including cerebellum) [81]. During the peak of forebrain neurogenesis (E10.5-E17), Shh regulates neural progenitor proliferation and differentiation in the germinal ventricular zone (VZ) [83], and it also affects interneuron generation and distribution in basal forebrain [24]. Shh signaling is necessary to maintain neurogenic niches in adult forebrain, including dentate gyrus (DG) and subventricular zone (SVZ) [50, 58, 74]. Shh produced by Purkinje cells (PCs) regulates granule cell precursor (GCP) proliferation, a process largely determining the cerebellum size and foliation that occurs mainly between E17.5 and postnatal day (P)15, and [21, 79].

Shh signaling deficiency causes birth defects [68, 84]. The Shh-null (Shh^−/−^) mouse is embryonic lethal with severe tissue patterning defects such as cyclopia, the most severe form of holoprosencephaly (HPE) [17, 72]. Chromosome abnormalities, including trisomy 13, 18, and 21, are the most frequent cause of HPE [46, 71]. Haploinsufficiency for Shh has little impact on gross phenotype in the mouse [39], but the loss of one allele of Shh is sufficient to cause HPE in humans [65]. Mice with both endogenous Shh alleles replaced with Shh^R34A/K38A^, mutations designed to inhibit the Shh-proteoglycan interaction, show reduced neural proliferation and growth defects (e.g., smaller body and cerebellar hypoplasia) [15]. Impaired Shh signaling has been seen in various neurological conditions including Down syndrome (DS) [23, 66], autism [10, 32], Alzheimer’s disease (AD) [40, 78], and Parkinson's disease (PD) [36, 70].

DS is the clinical result of trisomy for human chromosome 21 (HSA21) and occurs in ~ 1/800 new births [27]. Neurological features, including intellectual disability, early-onset dementia, and cerebellar hypoplasia, are ubiquitous in people with DS [2, 44]. Two population-based studies show that the prevalence of attention deficit hyperactivity disorder (ADHD) is significantly elevated in children with DS, which could be as high as 40% [28, 57]. Ts65Dn, the most widely used DS mouse model, recapitulates various DS brain phenotypes, including learning and memory deficits, hypocellular forebrain, disproportionally small cerebellum, and early degeneration of forebrain basal neurons [8, 18, 29, 60]. Locomotor hyperactivity in Ts65Dn has been consistently observed [29, 30, 33].

Trisomy in the Ts65Dn and other mouse models of DS attenuates the response to Shh in cerebellum, in neural crest forming the first pharyngeal arch, a precursor to craniofacial skeletal formation, and likely in other Shh-responsive cells and tissues [20, 66, 67]. This is not due to mutations in Shh or its canonical pathway proteins but results from overexpression of a gene or genes on HSA21 or its mouse orthologs. Cerebellar GCPs, which proliferate rapidly at birth in response to Shh, have a significantly reduced mitogenic response in trisomic compared to euploid (Eu) mice. Ts65Dn mice that recieved a single injection of a Shh signaling agonist (SAG) on the day of birth have normalized cerebellar morphology and improved learning and memory in adults [23].

To achieve brain region-specific Shh overexpression in vivo and test its protective or hazardous effects on cognitive function, we have created an inducible human Shh (hShh) knock-in mouse, TRE-bi-hShh-Zsgreen1 (TRE-hShh). We report that forebrain-specific Shh overexpression from the perinatal period not only normalizes locomotor hyperactivity and improves learning and memory in Ts65Dn mice but also enhances spatial cognition in Eu mice. In addition, cerebellum-specific Shh overexpression mitigates the cerebellar hypoplasia phenotype in Ts65Dn mice. These results provide the first in vivo evidence that Shh not only protects DS brain integrity but also enhances learning and memory in normal mice.

## Materials and methods

### Animal research

This study was carried out in accordance with the recommendations of the NIH Guide for the Care and Use of Laboratory Animals and the Johns Hopkins University (JHU) Institute of Animal Care and Use Committee. The protocol was approved by the Johns Hopkins University Institute of Animal Care and Use Committee. Mice were maintained in a JHU animal facility with 14-h light/10-h dark cycle, temperatures of 65–75°F (~ 18–23 °C) with 40–60% humidity, and fed with standard chow ad libitum and in-cage automatic water unless otherwise stated. Mouse models including Camk2a-rtTA, Pcp2-rtTA, Camk2a-tTA, Pcp2-tTA, TRE-hShh, and Gli1-LacZ, were backcrossed into C57BL/6 J background for more than 6 generations in our lab before any biochemical analysis or behavioral testing. Reporter strain TRE-LacZ mice (PMID 7,937,760, JAX 002,621) were a gift from Karen Hazzard (NIH/NHGRI). They were provided as cryopreserved embryos, recovered by the JHU Transgenic Core Laboratory, and maintained as a homozygous strain in SJL/J background throughout the project. Ts65Dn mice were maintained as an advanced intercross, C57BL/6 J × C3H/HeJ Fn (B6C3H). The details of genetic background and references are shown in the key sources table (Additional file 4: Table S1). Mouse colony management was supported by Softmouse (Iseehear).

ARRIVE guidelines (https://arriveguidelines.org/) were followed in the design and execution of the project. All experimental mice were generated through natural matings. A consolidated table of demographic animal information for each experiment of each figure, including genetic background, age, gender, and animal number, is provided (Additional file 5: Table S2). For biochemical analysis of animal tissues, mice/tissues were genotyped and assigned with unique identifiers. Investigators were blind to sample genotypes in all assays. Each cohort of mice was randomized before testing. Investigators of behavior tests were blind to mouse genotypes.

For genotyping, a mouse tail tip was placed in a 1.5 ml tube containing 600 ul of lysis buffer (50 mM Tris pH8, 100 mM EDTA, 0.5% SDS, and 400 mM NaCl) with 15 ul of 20 mg/ml Proteinase K (ThermoFisher) and incubating at 55 °C overnight. The tube was added 180 ul the saturated NaCl solution, mixed well, and then centrifuged at 13,000 rpm for 10 min at 4 °C. The supernatant was transferred to another 1.5 ml tube filled with 700 ul 100% EtOH, gently mixed to precipitate DNA, and then centrifuged at 13,000 rpm for 10 min at 4 °C. The DNA pellet was mixed with 500ul 70% EtOH, centrifuged at 13,000 rpm for 10 min at 4 °C, and dried and resuspended in 500 ul DEPC treated H_2_O. All genotyping primer sequence information is found in Additional file 6: Table S3.

### Construction of the targeting vector, pBT378-TRE-bi-hShh-Zsgreen1

The full-length hShh cDNA was amplified from HsCD00082632 (DNASU) with primers of Shh-halfRV and Shh-Bam2 and digested with BamHI and then ligated with pTRE3G-bi-ZsGreen1 (TaKaRa) after BamHI/EcoRV digestion to generate the first intermediate plasmid, pTRE3G-bi-hShh-Zsgreen1. The pTRE3G-bi-hShh-Zsgreen1 vector was digested with PciI and filled in Klenow, followed by EcoRI digestion to create the TRE-hShh cassette, which was ligated with pBS-SK( +) after SmaI/EcoRI digestion to create pBS-SK( +)-TRE-hShh. Zsgreen1/polyA, amplified from pTRE3G-bi-ZsGreen1 with primers of ClaI-F_Zs2 and RI-R_Zs, was ligated with pBS-SK( +)-TRE-hShh after ClaI/EcoRI digestion to generate the second intermediate plasmid, pBS-SK( +)-TRE-bi-hShh-Zsgreen1. The pBS-SK( +)-TRE-bi-hShh-Zsgreen1 vector was digested with ClaI and Not I to recover the fragment of TRE-bi-hShh-Zsgreen1 with a polyA signal. This was ligated with pBT378 after ClaI/Not I digestion to generate the targeting vector, pBT378-TRE-bi-hShh-Zsgreen1 (referred to herein as TRE-hShh targeting vector, Additional file 11: Figure S1A).

### Generation of TRE-hShh mice using TARGATT™ site-specific knock-in technology

In the JHU Transgenic Core Laboratory, 50 ng/ul φC31mRNA and 3 ng/ul TRE-hShh targeting vector were diluted in RNAse free injection buffer (10 mM Tris–HCl, pH 7.4, 0.25 mM EDTA) and injected into the pronucleus of 297 zygotes from Rosa26 TARGATT mice (Applied StemCell). The embryos were transferred into the oviducts of 11 pseudopregnant ICR moms. Founder pups were identified by PCR of tail DNA using primer sets SSL and SSR (Applied StemCell) that were specific for the right and left junctions of the attP/Rosa26 locus. Three mice positive for the insertion were identified among 31 pups. Two lines had the expected attP1/attP3 insertions, while the third contained only the attP3 insertion.

We verified founders using Southern blot analysis. The 5’- and 3’- probes were made by PCR with primer sets Tsh5prF/Tsh5prR of WT genomic DNA and Tsh3prF2/Tsh3prR3 of pROSA26-PA (plasmid #21,271, Addgene), respectively, and then labeled with α-32P-dCTP (Amersham Rediprime II Random Prime Labelling System). Genomic DNA from the 3 founder lines and controls were digested with HindIII, separated on 1 × TBE 0.9% agarose gel at 50 V overnight with recirculation, transferred to Hybond N + , and hybridized with 5ʹ- or 3ʹ- probes. The membrane was imaged on Molecular Dynamics Storm Imager and then exposed to film.

### Mouse embryonic fibroblast (MEF) cell culture and transfection

E14.5 mouse embryos were transferred into a 50 ml tube with 30 ml sterile PBS. Heads and liver/organs were removed with sterile razor blade. The remaining tissue was rinsed with PBS, placed in one well of a 6 well plate, then dissociated into fine pieces, and digested in 3 ml of 0.25% trypsin–EDTA at 37˚C for 20 min. The cell solutions were then transferred into a 10 cm plate containing 10 ml of MEF culture medium (DMEM, 10% FBS, 0.1 mM β-mercaptoethanol, 50 U penicillin, and 50 μg/ml streptomycin) and transferred to 37˚C incubator overnight. The next day, the culture plates were replaced with a 12 ml fresh MEF culture medium, which was replaced with fresh medium every 2 days thereafter. MEF cells were co-transfected with CMV-rtTA (pCMV-Tet3G, Clontech) and the TRE-hShh targeting vector using Lipofectamine 3000 (ThermoFisher). After 16 h, Doxycycline (Dox, Sigma-Aldrich) was added at 0, 0.1,1 ug/ml for 32 h. Cells were analyzed by Taqman RT-PCR, western blot, and immunostaining. TaqMan probes are detailed in Additional file 6: Table S3.

### Doxycycline delivery to mice

To test the inducibility of Shh expression, Camk2a-rtTA;TRE-LacZ and Pcp2-rtTA;TRE-LacZ were generated by crossing TRE-LacZ with Camk2a-tTA and Pcp2-tTA, respectively. Doxycycline (dox) was administered as 625 mg/kg in the diet (Envigo), or at 2 mg/ml or 3.5 mg/ml Dox (Sigma-Aldrich) in drinking water containing 2% sucrose, or both in the diet and drinking water. Mice were treated under the described regimens (Additional file 7: Table S4) and stained with X-gal at P30.

### Gene expression analysis

For Taqman RT-PCR, total RNAs were extracted by Trizol (ThermoFisher) and Chloroform (MilliporeSigma), followed by RNeasy column purification (QIAGEN). Cell pellets or homogenized tissues were mixed with 500 ul Trizol/sample (1 ml for larger samples) and then 100 µl chloroform and incubated at room temperature for 10 min. The sample was then centrifuged at 13,000 rpm for 20 min at 4 °C, and the upper, aqueous phase was transferred to a new tube and mixed with 1 volume of 70% EtOH/DEPC H_2_O. The sample was transferred to the RNeasy Mini Kit column (QIAGEN) and processed according to the manufacturer's directions. The TaqMan Gene Expression Master Mix (ThermoFisher) and Taqman probes were used to perform quantitative RT-PCR using QuantStudio 6 Flex Real-Time PCR Systems (ThermoFisher).

For Western blot, cell pellets or homogenized tissues were lysed in 500ul NP-40 lysis buffer (50 mM Tris (pH 7.5), 0.1% NP-40, 100 mM NaCl, 1 mM MgCl2, 5 mM EDTA) supplemented with 1X protease inhibitor cocktail and 1X Halt phosphatase inhibitor cocktail on ice for 30 min. Cell lysates were sonicated for 10 pulses at level 1 with 10% output 3 times and then incubating on ice for another 20 min. Soluble lysates were obtained by centrifugation at 17,000 × g for 20 min at 4 °C, and the protein concentration was measured by BCA (ThermoFisher). Protein extracts were separated by SDS-PAGE and transferred to nitrocellulose membranes. The nitrocellulose membranes were probed with primary and secondary antibodies described in the key sources table and washed extensively with PBS-T (0.1% Tween-20 in PBS).

For immunostaining of cultured cells, cells were grown and transfected on poly-D lysine coated coverslips and fixed in 4% PFA for 15 min. The cells were incubated with blocking buffer (5% normal goat serum and 0.1% Triton X-100) for 1 h at room temperature, primary antibodies at 4 °C overnight (or room temperature for 2 h), with Alexa Fluor conjugated secondary antibodies (ThermoFisher) for 45–60 min, and incubated in DAPI solution (1ul original DAPI diluted in 50 ml PBS-T) for 10 min. Coverslips were mounted on slides with “ProLong Gold Antifade Mountant with DAPI” (ThermoFisher) and dried, and then imaged using Zeiss LSM800 GaAsP (Microscope Facility, Johns Hopkins School of Medicine).

### Histology

For X-gal staining of brain sections, mice were perfused with PBS and 4% PFA. Brains were isolated and kept in 4% PFA overnight and embedded into optimal cutting temperature compound (OCT compound) and sectioned at 30 um using a Leica Cryostat CM 3050S. The sections were mounted on the glass slides and dried. Slides were fixed in fixation buffer (2% paraformaldehyde, 0.02% glutaraldehyde, 2 mM MgCl2 in PBS) for 10 min at room temperature, washed in PBS for 10 min and in wash solution (5 mM EGTA, 0.01% Deoxycholate, 0.02% NP40, 2 mM MgCl2 in 0.1 M phosphate buffer) for 10 min, then immersed in X-gal staining solution (5 mM K_3_Fe(CN)_6_, 5 mM K_4_Fe(CN)_6_, 5 mM EGTA, 0.01% Deoxycholate, 0.02% NP40, 2 mM MgC12, 1 mg/ml X-gal) overnight at 37 °C in the dark. Slides were rinsed in PBS for 10 min and distilled water for 5 min. Some slides were counter-stained with nuclear fast red for 1 min, rinsed in distilled water once, washed in distilled water for 1 min, dehydrated, and mounted with DPX and cover glasses.

For immunostaining, frozen brain Sects. (30 or 40 um) were fixed in 4% PFA for 30 min and rinsed twice with PBS. The sections were permeabilized and blocked with blocking buffer (0.5% Triton X-100 and 10% goat serum in PBS) for 1 h. The sections were reacted with primary antibodies diluted in blocking buffer at 4 °C overnight or at room temperature for 2 h (negative control was not treated with primary antibodies), followed by 3 washes in PBS-T for 10 min each, and then stained with Alexa Fluor conjugated secondary antibodies diluted in blocking buffer for 1 h followed by 3 washes in PBS-T, 10 min each. The sections were incubated in DAPI solution (1ul DAPI diluted in 50 ml PBS-T) for 10 min and then mounted on slides with “ProLong Gold Antifade Mountant with DAPI”. The Slides were kept in the dark overnight at room temperature and imaged using Zeiss LSM800 GaAsP. To create movies from confocal images, Z-stack and tile scan images were stitched in Zen (Zeiss) and converted to a movie in Imaris (Bitplane).

### Flow cytometry

Mice were deeply euthanized with isoflurane and perfused with cold PBS. Cerebellum, hippocampus, and cerebral cortex were removed, and cells were dissociated using Papain Dissociation System (Worthington-biochem) as described by the manufacturer.

The levels of Zsgreen1 expression for transgenic mice were analyzed using an SH800 Cell Sorter (Sony Biotechnology Inc, San Jose, CA). Cells were illuminated with a 488-nm laser, and fluorescence intensity was determined using the FL1 525 ± 50 nm emission filter. The minimal cell count of each sample was 200,000. Using FlowJo software v.10.1r7 (Ashland, OR), the same GFP-positive gating was used to separate GFP positive and negative cells for each group. The GFP positive (GFP +) percentage was calculated.

### MALDI-TOF MS analysis

The purity and concentration of recombinant Shh-N protein including recombinant mouse Shh-N (bacterial-derived, Cys25-Gly198, with a C-terminal 6-His tag, R&D 461SH), human Shh-N (bacterial-derived, an N-terminal Ile-Val-Ile sequence substituted for the naturally occurring Cys25 residue, PeproTECH 100–45), and human Shh-Np (HEK293-derived, human protein Cys24-Gly197 with C-terminal cholesterol and N-terminal fatty acid-modification, R&D 8908-SH/CF) were confirmed by Western blot and Coomassie blue staining. To make the matrix solution, 10 mg sinapinic acid was dissolved in 1 mL of 50:50 water/acetonitrile containing 0.1% trifluoracetic acid. 1 µL matrix was added to the MTP 384 target plate (Bruker) and dried, followed by depositing 1 µL protein, and then added another 1 µL matrix to the plate and dried. The MALDI-TOF spectra were acquired on a Bruker AutoFlex III (Billerica, MA) using positive ion mode.

### Behavior tests

3-month-old male mice of the Camk2a-promoted “forebrain-cohort” (Eu;TRE-hShh (n = 16), Eu;Camk2a-tTA;TRE-hShh (n = 14), Ts65Dn;TRE-hShh (n = 15), and Ts65Dn;Camk2a-tTA;TRE-hShh (n = 14)) and of the Pcp2-promoted “cerebellum-cohort” (Eu;TRE-hShh (n = 21), Eu;Pcp2-tTA;TRE-hShh (n = 20), Ts65Dn;TRE-hShh (n = 19), and Ts65Dn; Pcp2-tTA;TRE-hShh (n = 18)) were used for behavioral tests in the sequence, open field, visual discrimination water maze test, MWM, and RRWM. The ANY-maze tracking system (Stoelting Co.) was used to collect data.

After three days of handling in the same room, the open field tests were performed with indirect diffusing light (~ 150 l×). The whole arena size was 37 cm × 37 cm, and the center area (21.6 cm × 21.6 cm) was 34% of the whole arena. Distance traveled and the percentage of time spent in the center were analyzed. One Ts65Dn;TRE-hShh mouse in the forebrain-cohort and one Eu; Pcp2-tTA;TRE-hShh of the cerebellum-cohort were excluded from statistical analysis because of bad tracking.

For visual discrimination (VD), all mice were pre-trained to climb and stay on a submerged platform (10 cm × 10 cm) in a small clear water pool (45 cm diameter) for five trials on the day before VD tests. In a water tank 126 cm in diameter, non-toxic white tempera paint was used to make the platform invisible. No spatial cue was used, but the platform's location was made visible by attaching a black extension that was 4 cm above the water surface. During VD tests, the platform position started from W and to E and then to S, and two trials were performed in each platform position.

For classic MWM, the platform remained in the same position in the “SE quadrant” with the water temperature at 22 ± 2 °C. With the same spatial cues, MWM tests were performed for four training days. Each training day had 10 trials that included 8 acquisition trials and 2 probe trials of short- (30 min) and long- delay (24 h), and the longest delay probe trial was conducted on day 7, 72 h after the last trial of the training day 4. The platform was hidden ~ 1.8 cm below the water surface in acquisition trials, and 60 s maximum time was allowed for mice to find the platform. The tester would visually or manually guide it to the platform if a mouse did not find the platform by itself. In 30–40 s probe trials, the platform was lowered to a position that mice could not climb onto. At the end of probe trials, the collapsed platform was raised to the same position used in the acquisition trial, and the tester guided the mouse to the platform, which helped mice maintain the same response-reinforcement contingency of the acquisition. The quadrant target area, a circle inscribed in the platform quadrant, covered ~ 17% of the water maze tank. Trial 5 of Day 1 was a probe trial drill, during which the platform was lowered to a position that mice were not able to climb onto. Mice were only allowed to swim for 10 s, and then a tester raised the platform and guided mice to the platform, and no data from the trial were included for analysis. If a mouse continually failed to follow the tester’s guidance to reach the platform, it was to be excluded from analysis; no mice were excluded for this reason.

Following the classic MWM, the forebrain-cohort containing 12 Eu;TRE-hShh, 12 Eu; Camk2a-tTA;TRE-hShh, 10 Ts65Dn;TRE-hShh, and 10 Ts65Dn; Camk2a-tTA;TRE-hShh were tested in RRWM without changing any spatial cues. RRWM consisted of two reversal WM tests. In the first reversal WM, the platform was relocated to NW from SE for two training days. In the second reversal WM, the NW platform was relocated to SW for another two training days. Each training day had 10 trials, including 8 acquisition and 2 probe trials for short delay (30 min) and long delay (24 h). Trial 1 of reversal WM day 1 was the same as the 72 h delay probe trial in the classic MWM. This cohort was re-tested in MWM at ~ 7-month-old, which had the same protocol as the classic MWM above. The only differences were that the water tank was in a different room with different spatial cues.

### Brain morphometry by 3D T2-weighted MRI

The 14-month-old cerebellum-cohort (n = 8 per group) was used for ex vivo MRI. Mice were perfused with 4% PFA after PBS, and heads were post-fixed for 1 week, then kept in PBS for 3 days. Heads were stored in Fomblin to prevent dehydration during imaging in an 11.7 Tesla scanner (vertical bore, Bruker Biospin, Billerica, MA). 3D T2-weighted images were acquired on an 11.7 Tesla Bruker scanner (Bruker Biospin, Billerica, MA, USA) with the resolution = 0.08 mm × 0.08 mm × 0.08 mm, which were first aligned to the template image using automated image registration software (Diffeomap, www.mristudio.org) and adjusted to an isotropic resolution of 0.0625 mm × 0.0625 mm × 0.0625 mm. The region of interest (ROI) of the whole brain and cerebellum was manually drawn, and the whole brain volume and cerebellar volume were calculated for each mouse. The ratio of cerebellum to brain from each group was statistically compared (Additional file 8: Table S5).

### Beta-Glo assay

P6 Mice (Eu;Gli1-LacZ and Ts65Dn;Gli1-LacZ littermates, or TRE-hShh;Gli1-LacZ and Camk2a-tTA;TRE-LacZ;Gli1-LacZ littermates, or TRE-hShh;Gli1-LacZ and Pcp2-tTA;TRE-LacZ;Gli1-LacZ littermates) were euthanized. Cerebellum, hippocampus, and cerebral cortex were dissected and flash frozen in liquid nitrogen and stored at − 80 °C. On the day of beta-Glo assay, tissues of the whole cerebellum or hippocampus were incubated with Reporter Lysis Buffer (Promega) and disrupted using TissueLyser LT (QIAGEN) at 4 °C. The homogenized tissues were sonicated at 20% output for 10 s, repeated 3 times. The cell lysis was incubating on ice for 20 min, followed by centrifugation at 17,000 × g for 20 min at 4 °C. The protein concentration of the soluble lysate was determined by BCA protein assay. All groups were diluted to the same concentration. 2 ul and 5 ul lysates of each sample were mixed well with 100 μl of Beta-Glo Assay Reagent (Promega) in 96-well plates. The samples were incubated for 30 min at room temperature, and then luminescence was measured with a Wallac 1450 MicroBeta (PerkinElmer). Luminescence was normalized by protein concentration (LCPS/ug), and the relative LCPS/ug of littermates were compared and analyzed by paired t-tests.

### Quantification and statistical analysis

We used GraphPad Prism to do all statistical analysis unless otherwise stated. Generally, we used paired or unpaired t-tests to compare differences between two groups and one-way ANOVA and post-hoc Tukey's multiple comparisons tests to compare differences among three or more groups with one independent variable. To analyze the significance of two independent variables and compare differences between three or more groups, we used two-way ANOVA and post-hoc Tukey's or Sidak’s multiple comparisons tests. Data were represented as mean ± SEM. All significance thresholds were set at *P* < 0.05 unless otherwise stated. All the detailed statistical analysis for each figure was available in consolidated tables (Additional file 9: Table S6).

## Results

### Perinatal Ts65Dn mice show reduced Shh signaling in forebrain and cerebellum

Shh signaling is essential for proliferation and differentiation of neuronal progenitors (NPs) in developing forebrain [24, 83] and cerebellum [21, 79], and it is also required to maintain adult neurogenic niches [50, 58, 74]. Signal transduction in Shh-responding cells, including NPs, is through activation of one or more of three Gli transcription factors (Gli1, Gli2, and Gli3) that regulate the expression of G1 cell cycle proteins such as cyclin D and N-Myc and the antiapoptotic protein, Bcl-2 [13, 84]. As Gli1 is the primary Gli transcriptional activator [55] and is a sensitive readout in Shh signaling [1], we used a Gli1-LacZ reporter mouse [6] to track changes of Shh signaling in Shh-responding cells. Because the LacZ inserted into the first coding exon (exon 2) of Gli1, LacZ expression mimics the endogenous Gli1 activity.

**Fig. 1:**
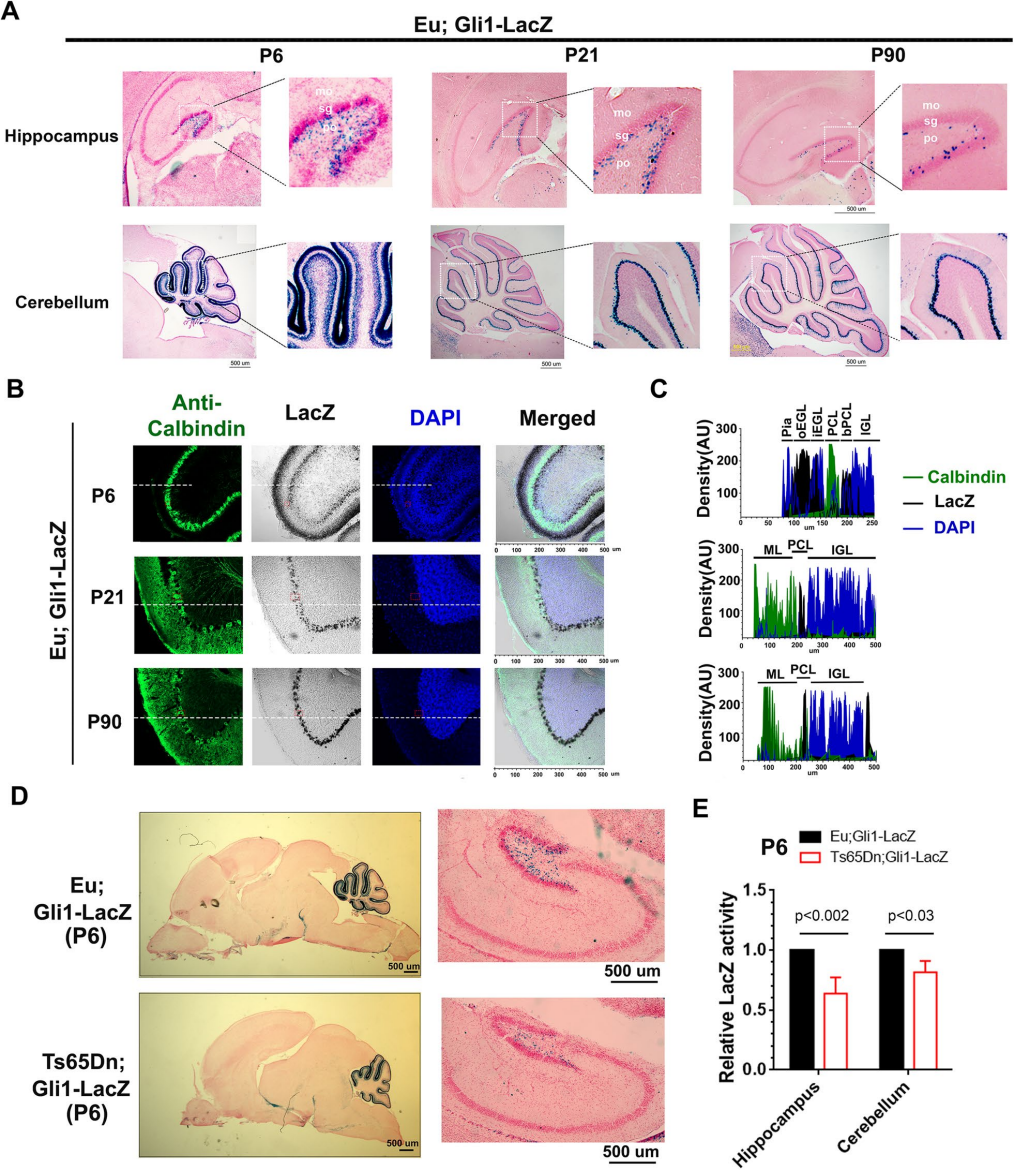
Ts65Dn shows reduced Gli1 activities in both hippocampus and cerebellum at P6. **A** X-gal staining of sagittal brain sections of Eu; Gli1-LacZ at P6, P21, and P90. Hippocampus and cerebellum areas were shown. mo, molecular layer; sg, subgranular zone; po, polymorphic layer. Scale bar = 500 um. **B** Representative images of P6, P21, and P90 of Eu; Gli1-LacZ cerebellum costained for LacZ (black) and Calbindin (green). Nuclear DNA was stained with DAPI (blue). **C** Detailed analysis of Gli1-positive cells in perinatal and adult cerebellum. Colocalization analysis of white dash labeled areas in (**B**). At P6, 6 distinct cerebellar cortical layers were found, and from outermost to innermost are Pia (high DAPI, LacZ negative, and Calbindin negative), oEGL (low DAPI, high LacZ, and Calbindin negative), iEGL (high DAPI, low LacZ, and Calbindin negative), PCL (low DAPI, LacZ negative, and Calbindin positive), bPCL (medium DAPI, medium LacZ, and Calbindin negative), and IGL (high DAPI, LacZ negative, and Calbindin negative). P21 or P90 cerebellum has three layers, ML (molecular layer), PCL, and IGL. N ≥ 3 per group (**A**–**C**). **D** X-gal staining of P6 sagittal brain sections of Eu;Gli1-LacZ and Ts65Dn;Gli1-LacZ. Hippocampus and cerebellum areas were compared, and n = 3 per group. **E** Beta-Glo assay of isolated P6 hippocampus and cerebellum from Eu; Gli1-LacZ and Ts65Dn;Gli1-LacZ. Data were represented as mean ± SEM and analyzed by two-way ANOVA and Sidak’s multiple comparisons tests, and n = 4 per group

In hippocampus of Gli1-LacZ mice, LacZ-positive cells were mainly located in the polymorphic layer and sub-granular layer of DG and significantly reduced from P6 to P21 to P90 (Fig. 1A). Cerebellar sections of P6, P21, and P90 Gli1-LacZ mice were costained with X-gal and anti-Calbindin immunostaining (Fig. 1B, C). At P6, most LacZ-positive cells were in outer external granular layer (oEGL) that contained GCPs, and a small population of LacZ-positive cells was located beneath the Purkinje cell layer (bPCL). By P21, LacZ-positive cells in the oEGL had disappeared, while LacZ-positive bPCL remained and was merging into PCL. At P90, LacZ-positive cells were completely integrated into PCL. Previous studies have linked Shh responding cells in adult PCL to Bergmann glia, a multi-functional astrocyte retaining neural precursor properties [43, 75].

X-gal staining showed that Ts65Dn;Gli1-LacZ mice had reduced LacZ levels in both hippocampus and cerebellum at P6 (Fig. 1D). Quantitative measurements of LacZ levels using Beta-Glo assay showed that LacZ activities were decreased by ~ 40% in hippocampus and by ~ 20% in cerebellum of P6 Ts65Dn;Gli1-LacZ (Fig. 1E), which indicates that perinatal Ts65Dn mice have a reduced Shh signaling in forebrain and cerebellum.

### The generation of an inducible hShh knock-in mouse, TRE-hShh

Shh precursor protein is autoproteolytically cleaved into Shh-N and Shh-C. The fully processed Shh-N known as “Shh-Np” is dually lipidated by attaching cholesterol to its C-terminus and palmitate to its N-terminus. The dual lipidation is essential to biological functions of Shh-Np, such as potency and long-range transport capacity [16, 19, 47, 61, 86]. Previous efforts have been made to produce mice with in-frame loxP or GFP modifications as markers of Shh expression. However, mice homozygous for these Shh modifications are embryonic lethal [14, 49]. To track Shh-overexpressing cells without disrupting Shh-Np function, we created the targeting vector “TRE-bi-hShh-Zsgreen1 (TRE-hShh)”, which was designed to simultaneously and independently express Zsgreen1 and hShh when transactivator (tTA) was present (Additional file 11: Figure S1A). We used TARGATT mice with 3 attP sites within the Rosa26 locus on chromosome 6 [72] to achieve site-specific integration of a single-copy transgene and avoid effects of random transgene integration. As the bacterial backbone (BB) could significantly reduce adjacent transgene expression in transgenic mice [73], genes of interest in the TRE-hShh vector were flanked by two attB sites to increase the chance of the transgene integration without BB.

**Fig. 2:**
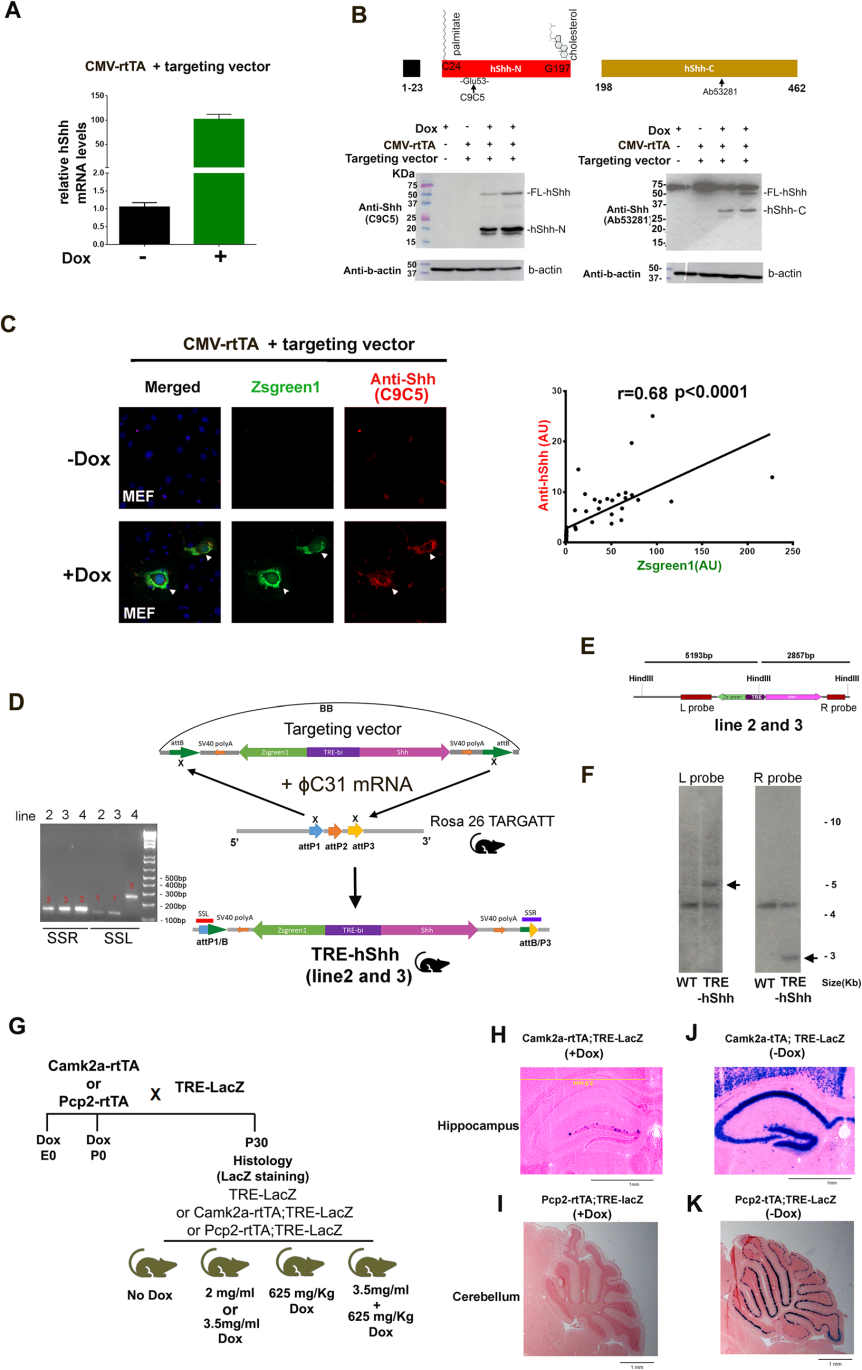
Generation of TRE-hShh transgenic mouse and verification of forebrain and cerebellum specific driver lines. **A** Taqman RT-PCR of MEFs co-transfected with CMV-rtTA and TRE-hShh vectors that were treated with or without Dox. Relative levels of hShh mRNA were compared, and beta (b)-actin was used for RT-PCR normalization. **B** Western blot of MEFs co-transfected with CMV-rtTA and TRE-hShh vectors that were treated with or without Dox. C9C5 (anti-Shh N-terminus antibody) and Ab53281 (anti-Shh C-terminus antibody) were used, and their targeting sites were shown in the top panel. **C** Immunostaining of MEFs co-transfected with CMV-rtTA and TRE-hShh vectors that treated with or without Dox. The left panel, representative confocal images (DAPI (blue), Zsgreen1 (green), and anti-hShh (red)).The right panel, the correlation between Zsgreen1 and anti-Shh. **D** Scheme for generating TRE-hShh transgenic mice through site-specific integration. ϕC31mRNA and the targeting vector were co-injected into the pronucleus of TARGATT mouse ES cells. Transgene insertions of three transgenic lines were analyzed by PCR using site-specific primer sets, SSL and SSR: SSL identified the left junction of attP, and 136 bp, 206 bp, and 282 bp PCR products indicated 5’ insertion at attP1, attP2, and attP3, respectively; SSR identified the right junction of attP, and 225 bp and155bp PCR products indicated 3’ insertion at attP2 and attP3, respectively. Line 2 and 3 had identical attP1/attP3 insertions with gene contents flanked by attB (without BB). Line 4 had attP3/attP3 insertion with the whole targeting vector (see Additional file 11: Figure S1C for details). **E** The scheme to predict southern blot products from TRE-hShh mice (line 2 and line 3). HindIII digestion producing two fragments: a 5193 bp left fragment (detectable with L probe that made of PCR products using Tsh3prF2/Tsh3prR3 primers) and a 2857 bp right fragment (detectable with R probe that made of PCR products using Tsh5prF/Tsh5prR primers). **F** Southern blots of WT and TRE-hShh (line2) using radiolabeled L and R probes. **G** The strategy of generating TRE-LacZ, Camk2a-rtTA;TRE-LacZ, and Pcp2-rtTA;TRE-LacZ mice to test rtTA driver lines. Mice were treated Dox from conception (E0) or P0 with various Dox administrations. All brain slices were stained with X-gal at P30, and n ≥ 5 mice per group. **H** Representative images of X-gal stained coronal brain sections from P30 Camk2a-rtTA;TRE-LacZ mice with Dox treatment (625 mg/Kg food pellets and 3.5 mg/ml in drinking water) from E0. Hippocampus was shown. Negative controls, including TRE-LacZ(+ Dox) and Camk2a-rtTA;TRE-LacZ (-Dox), were shown in Additional file 11: Figure S1F. **I** Representative images of X-gal stained sagittal brain sections from Pcp2-rtTA;TRE-LacZ mice with Dox treatment from E0 (625 mg/Kg food pellets and 3.5 mg/ml in drinking water). Cerebellum was shown. **J** Representative images of X-gal stained coronal brain sections from P30 Camk2a-tTA;TRE-LacZ mice without Dox treatment. Hippocampus was shown, and see Additional file 11: Figure S1G for the full sagittal section, and n ≥ 3. **K** Representative images of X-gal-stained sagittal brain sections from P14 Pcp2-tTA;TRE-LacZ mice without Dox treatment. Cerebellum was shown, and see Additional file 11: Figure S1H for the full sagittal section, and n ≥ 3. All brain slices in (**H**–**K**) were counter-stained with nuclear fast red. See also Additional file 11: Figure S1.

To test the TRE-hShh targeting plasmid that contains human Shh cDNA, mouse embryonic fibroblast (MEF) cells that do not contain any human genes were used for transfection. The real-time RT-PCR with a human Shh specific TaqMan probe showed that MEFs without plasmid transfection had a cycle threshold value > 40, indicating superior specificity of the probe (Additional file 10: Table S7). MEFs co-transfected with CMV-rtTA and TRE-hShh vectors showed little or no hShh transcription in the absence of doxycycline (Dox) and a prominent induction in its presence (Fig. 2A and Additional file 10: Table S7). Most full-length hShh was cleaved into hShh-N and hShh-C based on antibodies directed to either the N-terminus (C9C5 and 5H4) or C-terminus (Ab53281) of Shh, while MEFs expressed little or no endogenous Shh protein (Fig. 2B and Additional file 11: Figure S1B). Immunostaining of co-transfected MEFs with the C9C5 Shh antibody showed that only cells treated with Dox were Zsgreen1-positive and that Zsgreen1 fluorescent intensity was positively correlated with anti-Shh signal (r = 0.68 and *P* < 0.0001, Fig. 2C).

The TRE-hShh targeting vector and ϕC31 integrase mRNA were co-injected into TARGATT mouse ES cells to generate a TRE-hShh knock-in mouse. PCR for hShh and Zsgreen1 found that 3 of 15 pups had the targeting vector insertion. Using site-specific primer sets, SSL and SSR, we identified that founder line 2 and 3 had an identical attP1/attP3 transgene insertion without BB (Fig. 2D). Founder line 4 had the whole plasmid (with BB) inserted at the attP3 site (Additional file 11: Figure S1C). Southern blots of wildtype (WT) and the three transgenic lines validated respective transgene insertions (Fig. 2E, F and Additional file 11: Figure S1D-E). All three TRE-hShh lines were backcrossed into C57BL/6 J (B6) for > 6 generations, and heterozygous or homozygous mice were viable and fertile.

### Camk2a-tTA and Pcp2-tTA drive TRE-LacZ expression in forebrain and cerebellum, respectively

Expression of the Camk2a promoter is restricted to forebrain [76], and its activity begins at P1 and increases significantly by P5 [9]. The Pcp2 promoter drives expression in Purkinje cells (PCs) of cerebellum from E17.5 [48]. To find driver lines that could effectively induce TRE-hShh expression uniquely in forebrain or cerebellum, both Tet-on driver lines Camk2a-rtTA [51] and Pcp2-rtTA (unpublished), and Tet-off driver lines Camk2a-tTA [53] and Pcp2-tTA [89], were tested using a TRE-LacZ reporter mouse [35]. In the Tet-on system, attempts at activation with multiple modes and doses of Dox administration (1–3.5 mg/ml drinking water, 625 mg/Kg food pellet, or both, Fig. 2G) were not successful in inducing LacZ expression in Camk2a-rtTA;TRE-LacZ or Pcp2-rtTA;TRE-LacZ mice, even with continuous Dox exposure from conception (E0) (Fig. 2H, I). In contrast, Camk2a-tTA and Pcp2-tTA effectively drove TRE-LacZ expression in a forebrain- (Fig. 2J and Additional file 11: Figure S1G) and cerebellum- (Fig. 2K and Additional file 11: Figure S1H) specific manner, respectively.

### Camk2a-tTA;TRE-hShh mice overexpress dually-lipidated hShh-Np and enhance Shh signaling in forebrain

**Fig. 3:**
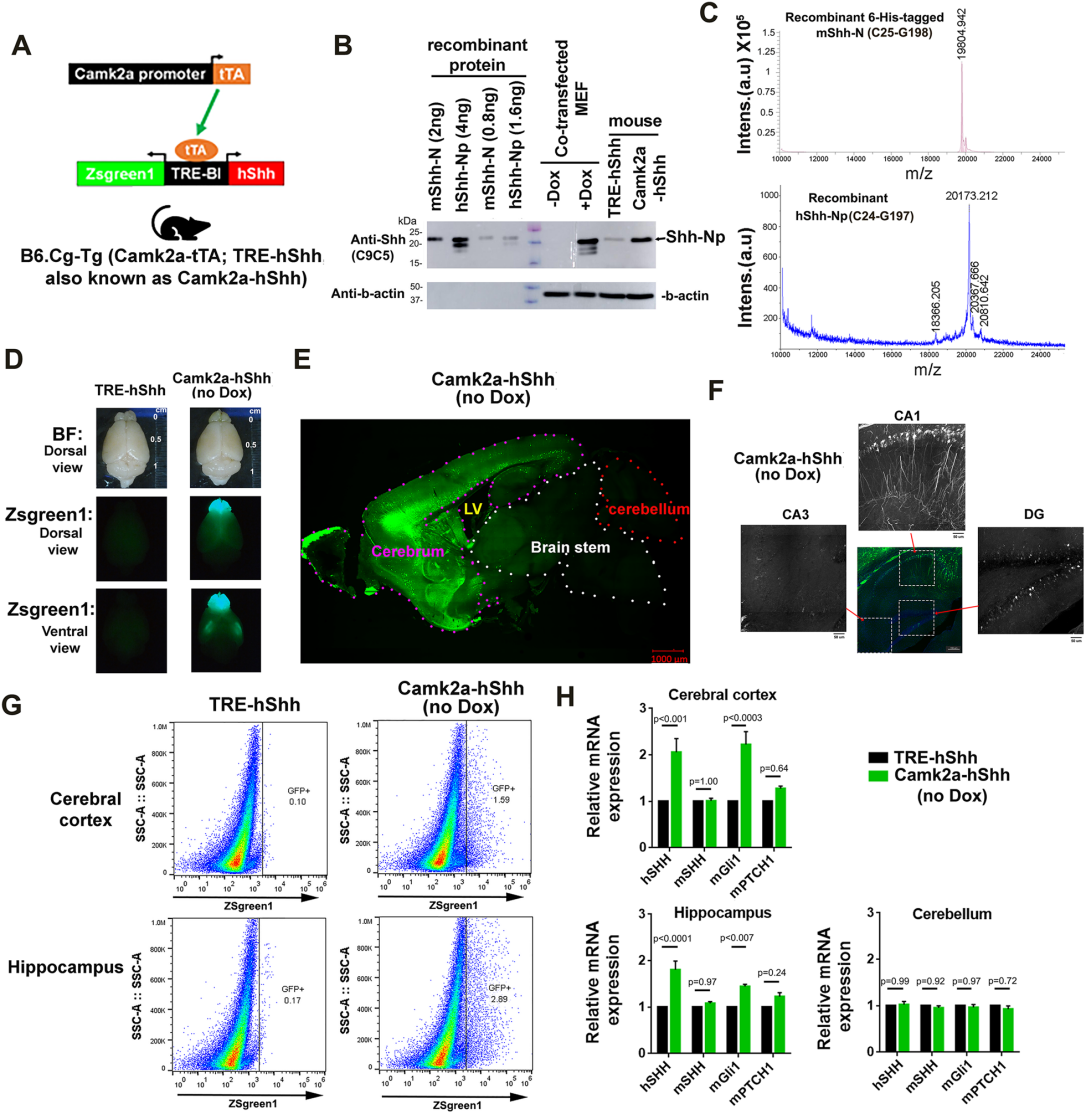
Camk2a-tTA;TRE-hShh mice overexpress dually-lipidated hShh-Np and enhance Shh signaling in forebrain. **A** Scheme of generating Camk2a-tTA;TRE-hShh mice. The active Camk2a promoter induces tTA expression that drives TRE-hShh to express Zsgreen1 and hShh. **B** Western blot of recombinant hShh-Np, 6xHis tagged mShh-N, lysate of MEF co-transfected with CMV-rtTA and the targeting vectors plus Dox treatment, and cerebral cortex lysate of TRE-hShh and Camk2a-hShh mice. The anti-hShh (C9C5) and anti-b-actin antibodies were used. **C** MALDI-TOF MS of recombinant protein. 6xHis tagged mShh-N (top) and hShh-Np (bottom). **D** Representative fluorescence microscopy images of TRE-hShh and Camk2a-hShh brains of 2-month-old mice. Both bright-field (BF) and GFP channels were shown. **E** Representative tile scan confocal images of a sagittal brain section of a 2-month-old Camk2a-hShh mouse. GFP channel was shown. **F** Zsgreen1-positive cells in Camk2a-hShh hippocampus. See Additional files 1 and 2: Video S1 and S2 for details. **G** FACS of dissociated cells from cerebral cortex and hippocampus of 2-month-old TRE-hShh and Camk2a-hShh mice. The same gate setting was used to separate Zsgreen1-negative and Zsgreen1-positive cells for all samples, and n = 2 per group. **H** Taqman RT-PCR of Shh signaling pathway genes (hShh, mShh, mGli1, and mPTCH1). Cerebral cortex, hippocampus, and cerebellum were compared. The b-actin was used for the RT-PCR normalization. Data were represented as mean ± SEM and analyzed by two-way ANOVA and Sidak’s multiple comparisons tests, and n = 5 per group. See also Additional files 1 (Video S1), 2 (Video S2), 11 (Figure S2).

B6.Camk2a-tTA were crossed with all three B6.TRE-hShh lines to generate double Camk2a-tTA;TRE-hShh (Camk2a-hShh) transgenic mice (Fig. 3A). Camk2a-hShh from lines 2 and 3 had Zsgreen1 expression patterns in brain similar to Camk2a-tTA;TRE-LacZ, while Camk2a-hShh from line 4 (with the bacterial backbone included at the insertion site) had little Zsgreen1 expression (Additional file 11: Figure S2A). TRE-hShh line 2 mice were used for subsequent experiments. Both recombinant hShh-Np purified from HEK293 cells (purchased from R&D Systems) and the lysates of CMV-rtTA and TRE-hShh co-transfected MEFs showed three ~ 20 kDa bands in Coomassie blue staining and Western blot, the highest molecular weight band was the most prominent (Fig. 3B and Additional file 11: Figure S2B). The hShh-N, the C24-G197 of Shh precursor protein, has a theoretical molecular weight (MW) of 19.56 kDa before post-translational modifications and palmitoylation at C24 and cholesterolization at G197 add ~ 238 Da and ~ 369 Da, respectively. MALDI-TOF mass spectrometry of recombinant hShh-Np showed the peak at ~ 20.173 kDa (Fig. 3C), which matches the theoretical MW of dually-lipidated hShh-Np. There is only one amino acid difference in Shh-N of the human and the mouse, wherein S44 of hShh-N is T44 in mShh-N (Additional file 11: Figure S2C). Unlike MEFs with transient overexpression of hShh, both Camk2a-hShh and control (TRE-hShh) mice only expressed the dually-lipidated Shh-Np form (Fig. 3B).

Camk2a-hShh pups at postnatal day 1 (P1) without Dox treatment were GFP-positive in forebrain and could be identified by GFP flashlight (Additional file 11: Figure S2E). In 2-month-old mice, no general brain morphology difference was found between TRE-hShh and Camk2a-hShh (Fig. 3D). Sagittal brain sections of Camk2a-hShh showed that forebrain structures, including olfactory bulb, cerebral cortex, hippocampus, and basal ganglia, were Zsgreen1-positive, while cerebellum and brain stem were Zsgreen1-negative (Fig. 3E). In the hippocampus, Zsgreen1-positive neurons were densely packed in DG and CA1 but were sporadic in CA3 (Fig. 3F and see Additional files 1, 2: Video S1 and S2 for details). Using a conservative gate setting for GFP-positive cells, fluorescence-activated cell sorting (FACS) of Camk2a-hShh showed Zsgreen1-positive cells accounted for ~ 1.6% of cortical cells and ~ 2.9% of hippocampal cells (Fig. 3G). Key transcripts of canonical Shh signaling pathway, including mShh, mPTCH1, and mGli1, were compared between TRE-hShh and Camk2a-hShh mice at 2-month-old (Fig. 3H). In cortex or hippocampus, hShh transcripts in Camk2a-hShh levels were twice as high as those in TRE-hShh. Based on the percentages of Zsgreen1-positive cells in FACS, we deduced that the presence of Camk2a-tTA could induce TRE-hShh expression by more than 50 fold. The mGli1 expression was significantly increased in cortex (*P* < 0.0003) and hippocampus (*P* < 0.007) of Camk2a-hShh. As endogenous mShh transcripts were not changed (*P* = 1.0 in cortex and *P* = 0.97 in hippocampus), we attributed the activation of Shh signaling in forebrain of Camk2a-hShh to hShh expression. Together, these results show that Camk2a-hShh enhances Shh signaling in forebrain through overexpression of the dually-lipidated Shh-Np form, which is essential to study Shh dosage effects.

### Pcp2-tTA;TRE-hShh mice show PC-specific hShh overexpression and enhance Shh signaling in cerebellum

**Fig. 4:**
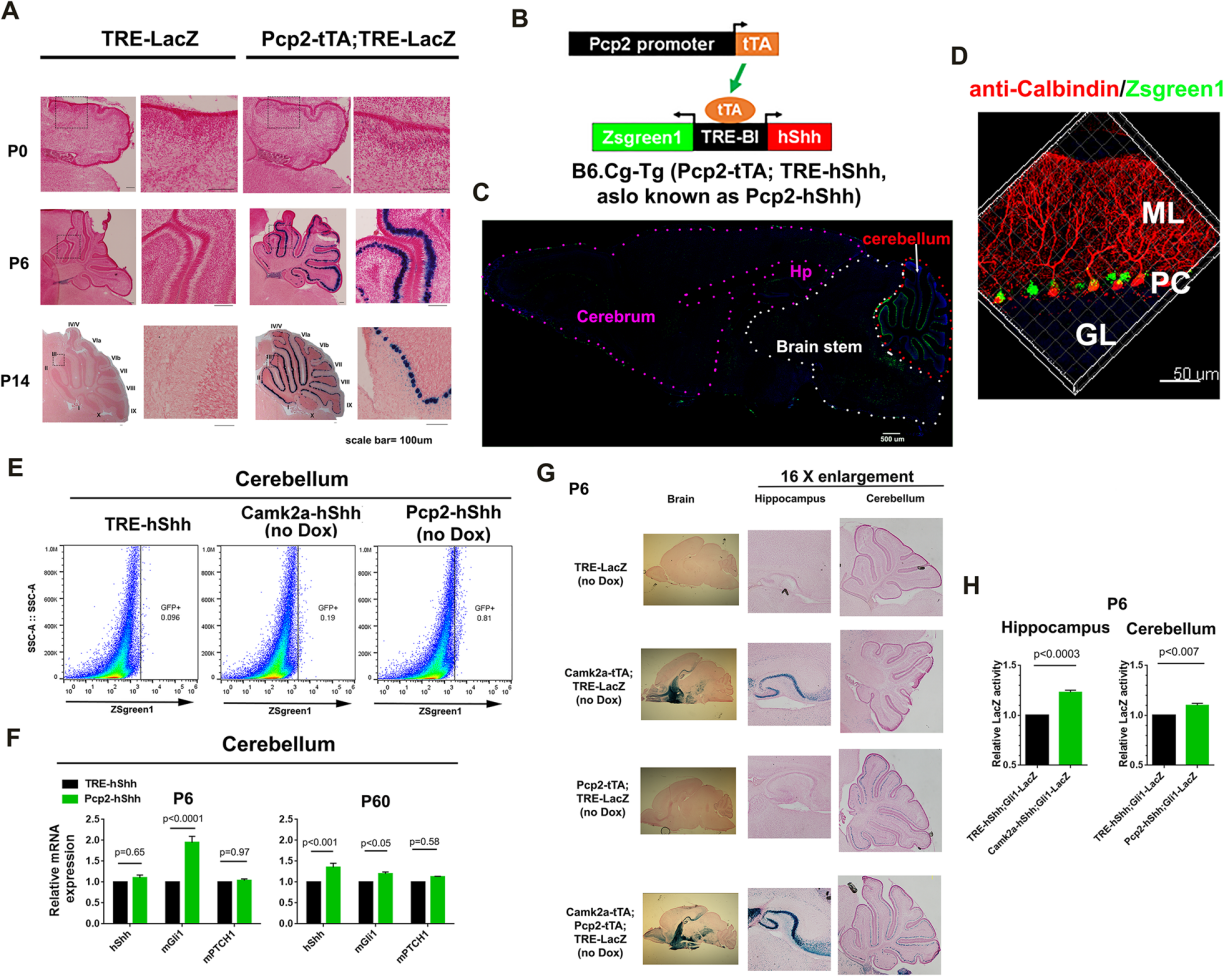
Pcp2-tTA;TRE-hShh mice show PC-specific hShh overexpression and enhance Shh signaling in cerebellum. **A** X-gal staining of sagittal brain sections of TRE-LacZ and Pcp2-tTA;TRE-LacZ at P0, P6, and P14. Brain sections were counter-stained with nuclear fast red, and cerebellum was shown. **B** Scheme of generating B6.Pcp2-tTA;TRE-hShh double transgenic mice. **C** Representative tile scan confocal images of a sagittal brain section of a 2-month-old Pcp2-hShh mouse. GFP channel was shown. **D** Immunostaining of Pcp2-hShh sagittal brain sections for Calbindin (red). Granular layer (GL), PC, and molecular layer (ML) were shown, and see Additional file 3: Video S3 for details. **E** FACS of cerebellums from TRE-hShh, Camk2a-hShh, and Pcp2-hShh mice. **F** Taqman RT-PCR for hShh, mGli1, and mPTCH1 from TRE-hShh and Pcp2-hShh cerebellum at P6 and P60. Data were represented as mean ± SEM and analyzed by repeated two-way ANOVA and Sidak’s multiple comparisons tests, and n = 5 per group. **G** Sagittal sections of P6 brains of TRE-LacZ, Camk2a-tTA;TRE-LacZ, Pcp2-tTA;TRE-LacZ, and Camk2a-tTA;Pcp2-tTA;TRE-LacZ mice were analyzed by X-gal staining. The sections were counter-stained with nuclear fast red. **H** P6 hippocampus from 5 pairs of “TRE-hShh;Gli1-LacZ and Camk2a-hShh;Gli1-LacZ” littermates and P6 cerebellum from 5 pairs of “TRE-hShh;Gli1-LacZ and Pcp2-hShh;Gli1-LacZ” littermates analyzed by Beta-Glo assay. Data were represented as mean ± SEM and analyzed by paired t-tests. See also Additional files 3 (Video S3), 11 (Figure S3).

The first postnatal two weeks are critical for mouse cerebellar development [34, 52], which is closely correlated with GCP proliferation that is induced by Shh produced by PCs. To validate that Pcp2-tTA could induce TRE promoter in PCs from the perinatal period, sagittal brain sections of P0, P6, and P14 Pcp2-tTA;TRE-LacZ mice were analyzed by X-gal staining (Fig. 4A). At P0, multiple layers of small and low-intensity LacZ-positive cells were detected, which matches PC aggregation in a primordial cortical layer of cerebellum at the stage [64, 69]. From P0 to P14, both the size and intensity of LacZ-positive cells increased, forming a single layer by P14. B6.Pcp2-tTA mice were crossed with B6.TRE-hShh to generate Pcp2-tTA;TRE-hShh (herein, Pcp2-hShh, Fig. 4B). Zsgreen1 expression was restricted in cerebellum of 2-month-old Pcp2-hShh mice (Fig. 4C), and Calbindin1 immunostaining confirmed that only PCs were Zsgreen1-positive (Fig. 4D and Additional file 3: Video S3). FACS of Pcp2-hShh brain structures showed Zsgreen1-positive cells in cerebellum but not in cortex or hippocampus (Fig. 4E and Additional file 11: Figure S3). Compared with TRE-hShh, Pcp2-hShh significantly increased mGli1 transcript levels in cerebellum, with the higher induction at P6 than P60 (Fig. 4F), which was likely due to more Shh responding cells (primarily GCPs) in developing cerebellum than in adult cerebellum.

Camk2a-tTA and Pcp2-tTA induced TRE-LacZ expression in P6 forebrain and cerebellum, respectively (Fig. 4G). To test whether Camk2a-hShh and Pcp2-hShh enhanced Shh signaling from the perinatal period, triple transgenic mice, Camk2a-tTA;TRE-hShh;Gli1-LacZ (Camk2a-hShh;Gli1-LacZ) and Pcp2-tTA;TRE-hShh;Gli1-LacZ (Pcp2-hShh;Gli1-LacZ), were generated and assessed at P6. Compared with P6 TRE-hShh;Gli1-LacZ littermates, Camk2a-hShh;Gli1-LacZ increased LacZ activity by ~ 25% in hippocampus and Pcp2-hShh;Gli1-LacZ increased LacZ activity by ~ 10% in cerebellum (Fig. 4H). These results indicate that Camk2a-tTA;TRE-hShh and Pcp2-tTA;TRE-hShh can increase Shh signaling in forebrain and cerebellum, respectively, from the perinatal period.

### Camk2-hShh but not Pcp2-hShh normalizes locomotor hyperactivity in Ts65Dn

**Fig. 5:**
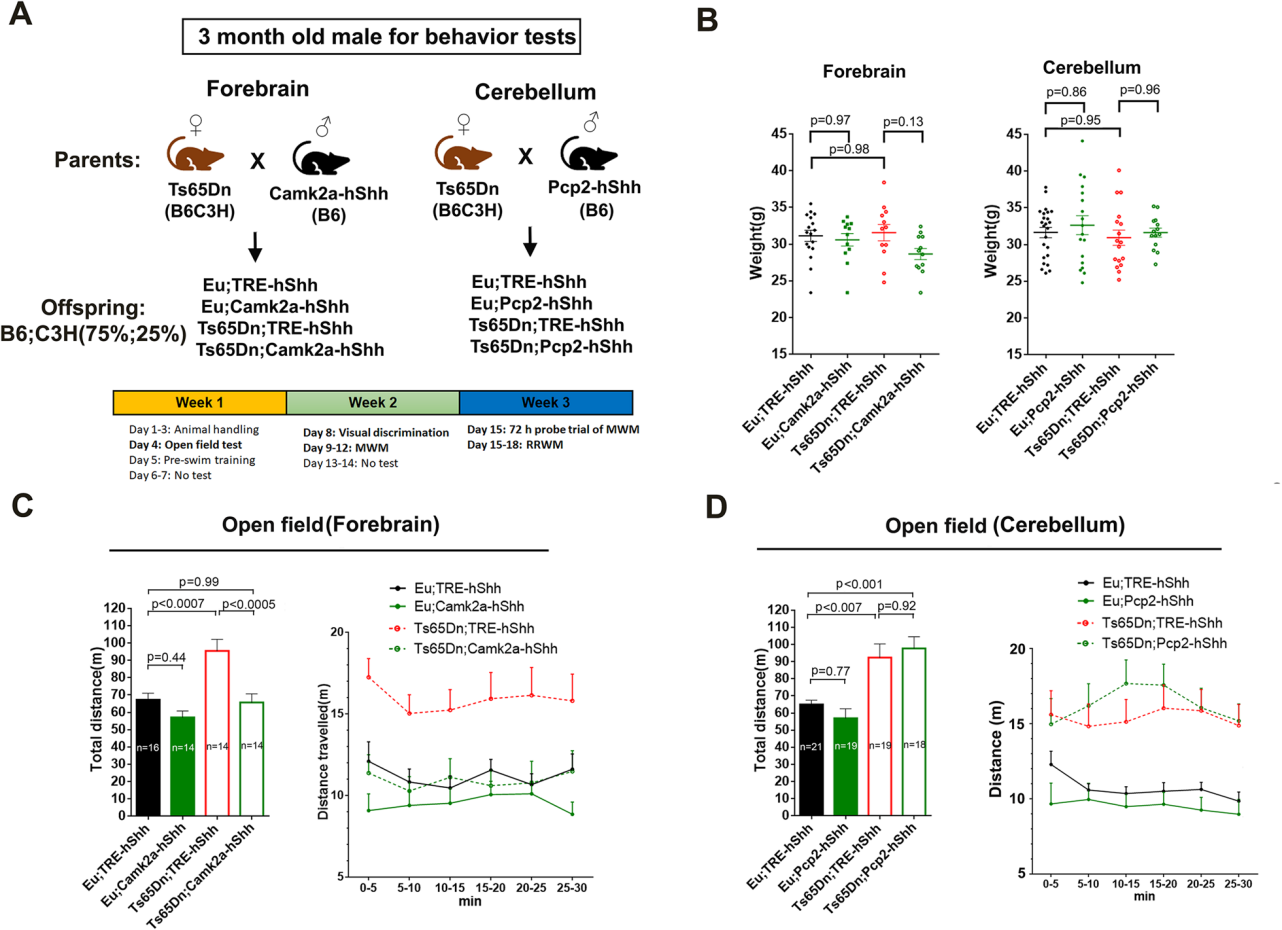
Camk2-hShh but not Pcp2-hShh normalizes locomotor hyperactivity in Ts65Dn. **A** Scheme for generating the forebrain-cohort (n = 14–16 per group) and the cerebellum-cohort (n = 18–21 per group) and the order of behavioral tests. All experimental animals were in B6;C3H (75%;25%) background and without Dox treatment. **B** Body weight of different transgenic mice at 3-month-old. **C** 30 min open field tests of the forebrain-cohort. Total distance traveled in 30 min (left) and distance traveled in each of 5-min-bin (right) were shown, and n = 14–16 per group. **D** 30 min open field tests of the cerebellum-cohort. Total distance traveled in 30 min (left) and distance traveled in each of 5-min-bin (right) were shown, and n = 18–21 per group. Data were represented as mean ± SEM. Data were analyzed by one-way ANOVA and Tukey's multiple comparisons tests (**B**). Total distances traveled during 30 min (the left panel of **C** and **D**) were analyzed by one-way ANOVA and Tukey's multiple comparisons tests. The distances traveled in each of 5-min-bin (the right panel of **C** and **D**) were analyzed by two-way RM ANOVA and Tukey's multiple comparisons tests. See also Additional file 11: Figure S4.

In single-transgenic lines, transgenes including Camk2a-tTA (*P* = 0.28), Pcp2-tTA (*P* = 0.94), and TRE-hShh (*P* = 0.61), were inherited in Mendelian proportions (Additional file 11: Figure S4A). When breeding for double transgenic mice, there were more Camk2a-hShh (*P* = 0.05) and Pcp2-hShh (*P* < 0.03) than TRE-hShh littermates (Additional file 11: Figure S4B). P40 Camk2a-hShh mice treated with Dox from E0 to E17 had significantly lower Zsgreen1 expression than those without prenatal Dox treatment (Additional file 11: Figure S4C), consistent with previous studies that Dox treatment during embryonic development may prevent the Tet-off system from achieving full activation in adult brain [45]. To induce hShh overexpression in Ts65Dn brain from the perinatal period and analyze its effects at its fullest potential, both “forebrain-cohort” (offspring of the cross between B6C3H.Ts65Dn females and B6.Camk2a-hShh males) and “cerebellum-cohort” (offspring of the cross between B6C3H.Ts65Dn females and B6.Pcp2-hShh males) were generated without Dox treatment. Thus, all experimental mice were in the B6;C3H (75%;25%) background. These mice were analyzed in behavioral tests, including open field, visual discrimination, and Morris water maze (MWM) at 3-month-old (Fig. 5A). The forebrain-cohort consisted of 14–16 males in each of four groups, including Eu;TRE-hShh, Eu;Camk2a-hShh, Ts65Dn;TRE-hShh, and Ts65Dn;Camk2a-hShh, and the cerebellum-cohort consisted of 18–20 males in each of four genotypes, including, Eu;TRE-hShh, Eu;Pcp2-hShh, Ts65Dn;TRE-hShh, and Ts65Dn;Pcp2-hShh. In 3-month-old mice, we confirmed that Camk2-hShh expressed significantly more Shh-Np in forebrain than TRE-hShh (Additional file 11: Figure S4D) and that Pcp2-hShh expressed significantly more Gli1 protein in cerebellum than TRE-hShh (Additional file 11: Figure S4E).

Shh overexpression in forebrain or cerebellum had no significant effect on body weight of Eu or Ts65Dn mice (Fig. 5B). Locomotor hyperactivity in Ts65Dn has been observed repeatedly since the model was established [29, 30, 33]. In a 30 min novel open field paradigm, forebrain Shh overexpression in Ts65Dn completely normalized locomotor hyperactivity based on the total distance traveled (*P* < 0.0003 for Eu;TRE-hShh VS Ts65Dn;TRE-hShh, and *P* = 0.95 for Eu;TRE-hShh VS Ts65Dn;Camk2a-hShh, Fig. 5C). Cerebellum Shh overexpression did not affect locomotor hyperactivity of Ts65Dn (*P* < 0.005 for Eu;TRE-hShh VS Ts65Dn;TRE-hShh, and *P* < 0.0007 for Eu;TRE-hShh VS Ts65Dn;Pcp2-hShh Fig. 5D).

### Camk2-hShh but not Pcp2-hShh improves learning and memory in Eu and Ts65Dn

The effects of Shh overexpression on visual ability and goal-directed behaviors were tested using visual discrimination (VD), a non-spatial learning water maze with a cued hidden platform (Additional file 11: Figure S5A). Ts65Dn performed slightly worse than Eu in VD (*P* = 0.08 in forebrain-cohort and *P* = 0.06 in cerebellum-cohort), while hShh overexpression in forebrain or cerebellum had no significant effect on the VD performance (Additional file 11: Figure S5B).

**Fig. 6:**
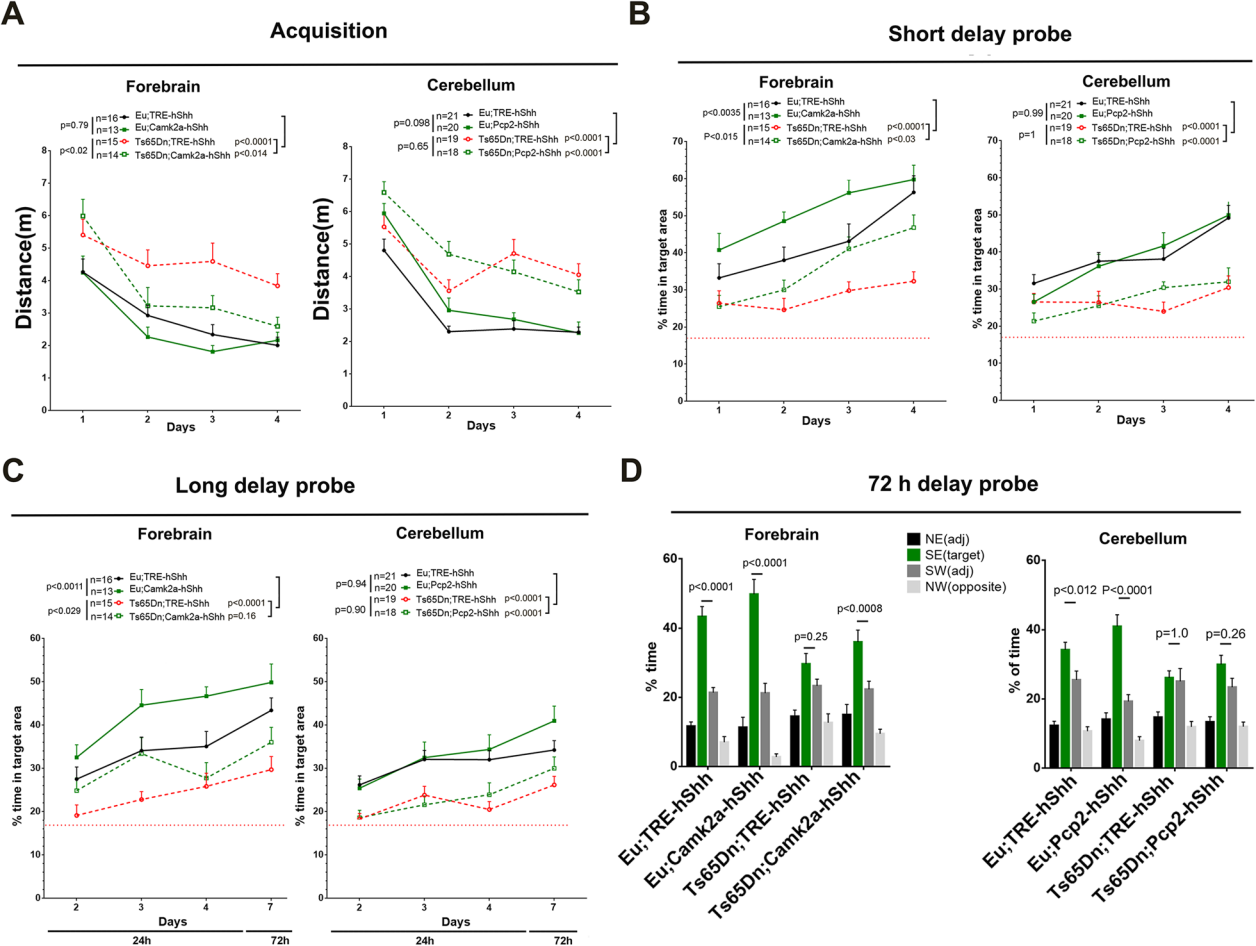
Camk2-hShh but not Pcp2-hShh improves learning and memory in Eu and Ts65Dn. **A** Escape distance in acquisition trials from training day (TD)1 to 4. **B** Short (30 min) delay probe trials from TD 1 to 4. **C** Long delay probe trials. TD2-TD4, 24 h delay probe trials; TD7, 72 h delay probe trials. **D** The quadrant preference analysis of the 72 h long delay probe trials. Data were represented as mean ± SEM and analyzed by two-way ANOVA and Tukey's multiple comparisons tests. N = 13–16 per group in the forebrain-cohort, and n = 18–21 per group in the cerebellum-cohort. See also Additional file 11: Figure S5 and S6.

We used a 4-day training MWM to assess forebrain or cerebellum Shh overexpression effects on spatial learning and memory (Additional file 11: Figure S6A). Because Ts65Dn swam faster than Eu in acquisition trials (*P* < 0.0001, Additional file 11: Figure S6B), the performance in acquisition trials was reported as escape distance rather than latency. As training progressed, escape distance improved in all groups, but the improvement was significantly slower in Ts65Dn than Eu (*P* < 0.0001, Fig. 6A). In the forebrain-cohort, forebrain hShh overexpression significantly reduced escape distance in Ts65Dn (*P* < 0.02). In the cerebellum-cohort, hShh overexpression in cerebellum had no significant effect on escape distance in Ts65Dn (*P* = 0.65).

Four-day overall performance in short delay probe trials (Fig. 6B) showed that Ts65Dn lagged significantly behind Eu (*P* < 0.0001), and that forebrain hShh overexpression significantly improved the performance in both Eu (*P* < 0.0035) and Ts65Dn (*P* < 0.015) and mitigated the deficiency caused by trisomy (*P* < 0.0001 for Eu;TRE-hShh VS Ts65Dn;TRE-hShh, *P* < 0.03 for Eu;TRE-hShh VS Ts65Dn;Camk2a-hShh), and that cerebellum hShh overexpression in cerebellum had no significant effect on Eu (*P* = 0.99) or Ts65Dn (*P* = 1.0).

In four-day overall performance in long delay probe trials (Fig. 6C), forebrain hShh overexpression significantly enhanced the performance in both Eu (*P* < 0.001) and Ts65Dn (*P* < 0.029) and rescued the trisomy-caused deficiency (*P* < 0.0001 for Eu;TRE-hShh VS Ts65Dn;TRE-hShh, *P* = 0.16 for Eu;TRE-hShh VS Ts65Dn;Camk2a-hShh). The quadrant preference analysis of 72 h long delay probe trials showed that forebrain Shh overexpression significantly improved the performance in Ts65Dn (Fig. 6D). Cerebellum hShh overexpression had no significant effect on the performance of long delay probe trials of Eu (*P* = 0.94) or Ts65Dn (*P* = 0.90).

### Camk2a-hShh improved the ability to forget non-essential memory

To assess the effects of forebrain Shh overexpression on the integrity of learning and memory (i.e., inhibition of non-essential memory and retention of essential spatial reference memory), we continued to test the forebrain-cohort in the repeated reversal water maze (RRWM, Additional file 11: Figure S7A) after the 72 h probe trial of MWM. In reversal session 1 (reversal (R) day1 and day2), the hidden platform was moved from SE to NW. The platform was then relocated to SW in reversal session 2 (R day3 and day4). Thus, the old platform position “SE” is the non-essential memory in RRWM, and the spatial reference is the essential memory as we used the same spatial reference in RRWM and MWM.

**Fig. 7:**
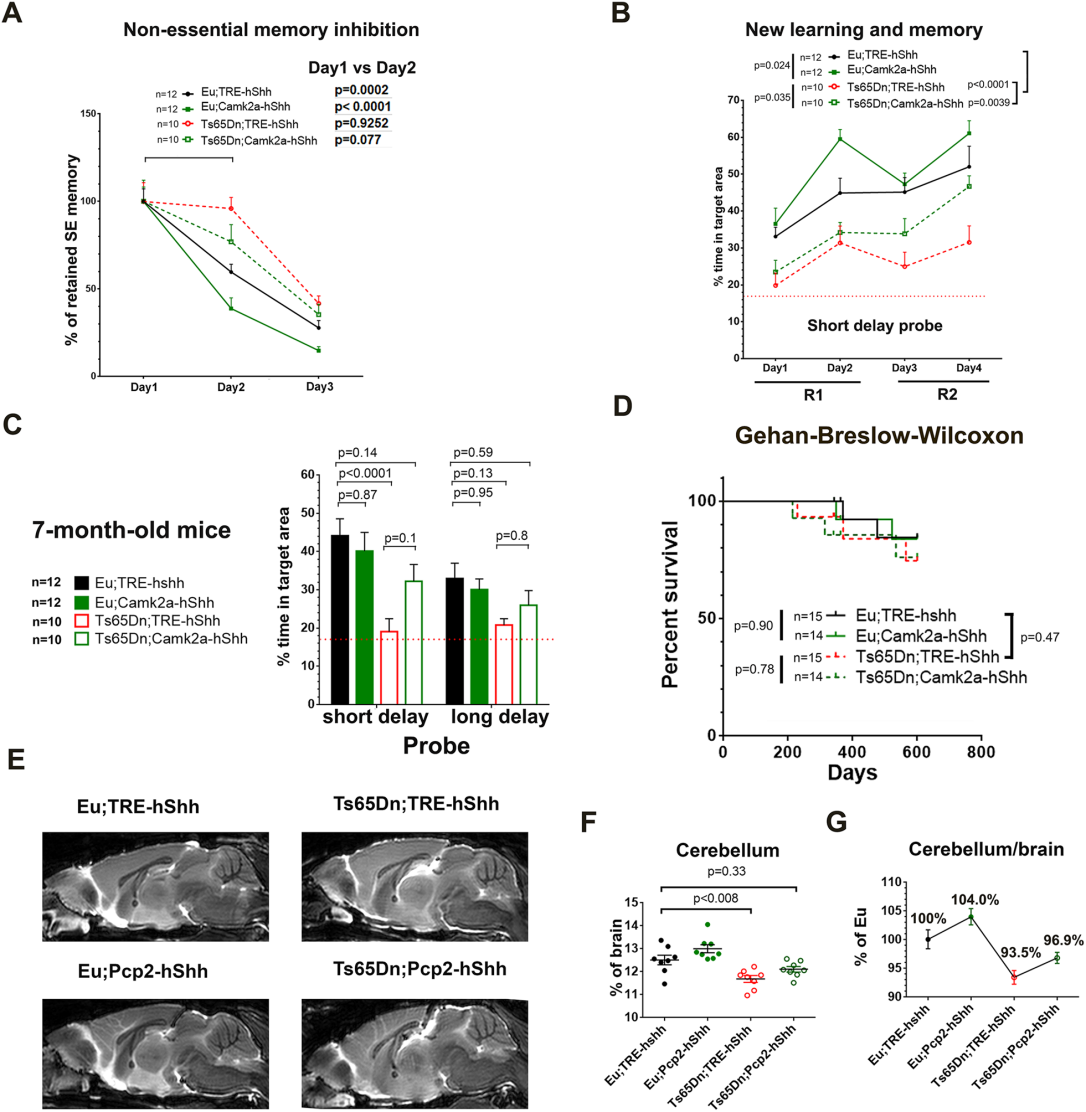
RRWM and survival analysis of the forebrain-cohort and MRI of the cerebellum-cohort. **A** RRWM of the forebrain-cohort. Long delay probe trials for the old platform position “SE” from day1 to day3, and n = 10–12 per group. **B** RRWM short delay probe trials for new platform positions. R, reversal session. **C** The forebrain-cohort was re-tested in MWM at 7-month-old (n = 10–12 per group). **D** Survival analysis of the forebrain-cohort aged until 600 days old (n = 14–15 per group). **E** Representative T2-weighted MRI of mid-sagittal brain of the 14-month-old cerebellum-cohort. **F** Statistical analysis of the cerebellum/brain volume ratio based on the MRI (n = 8 per group). **G** The cerebellum/brain ratio of other groups relative to Eu controls based on the MRI. Data were represented as mean ± SEM and analyzed by two-way ANOVA and Tukey's multiple comparisons test (**A**–**C**), and by Gehan-Breslow-Wilcoxon test (**D**), and by two-way ANOVA and Tukey's multiple comparisons tests (**F**–**G**). See also Additional file 11: Figure S7.

To quantify the ability to forget the non-essential memory, we compared % of time spent in the SE target area in long delay probe before RRWM training (trial 1 of R day1) with that after 1 day training (trial 1 of R day2) and after 2 days training (trial 1 of R day3, Fig. 7A). After 1 day RRWM training, the average SE memory reduction was 40% in Eu;TRE-hShh (*P* = 0.0002), 61% in Eu;Camk2a-hShh (*P* < 0.0001), 4% in Ts65Dn;TRE-hShh (*P* = 0.93), and 23% in Ts65Dn;Camk2a-hShh (*P* = 0.077). Probe trials showed that forebrain Shh expression significantly increased time spent in new target areas (*P* = 0.024 in Eu, *P* = 0.035 inTs65Dn, Fig. 7B).

### Camk2a-hShh delays early-onset severe cognitive impairment in 7-month-old Ts65Dn and does not affect longevity

People with DS have a high risk of developing early-onset dementia, and previous studies show that Ts65Dn mice lose the ability to acquire any spatial learning and memory in MWM at ~ 6-month-old [11, 12]. To analyze the effects of forebrain hShh overexpression on early-onset severe cognitive impairment in Ts65Dn, we tested the forebrain-cohort in an MWM decorated with different spatial references at 7-month-old. The short delay probe trials of day 4 training showed that the average percentage of time spent in the target area was 19% in Ts65Dn;TRE-hShh (close to the 17% chance level), 32% in Ts65Dn;Camk2a-hShh, and 41% in Eu;TRE-hShh (Fig. 7C). Forebrain hShh overexpression mitigated the trisomy-caused early-onset severe cognitive impairment in Ts65Dn (*P* < 0.0001 for Eu;TRE-hShh VS Ts65Dn;TRE-hShh, and *P* = 0.14 for Eu; TRE-hShh VS Ts65Dn;Camk2a-hShh, Fig. 7C).

To evaluate side effects of long-term hShh overexpression in forebrain, we aged the forebrain-cohort (14–15 per group) until 600 days old for survival analysis. The Gehan-Breslow-Wilcoxon analysis (Fig. 7D), giving more weight to early deaths, showed that the mortality rate of Ts65Dn was slightly higher than Eu (*P* = 0.47) and that chronic hShh forebrain overexpression showed no significant effect on longevity in Eu (*P* = 0.90) or Ts65Dn (*P* = 0.78).

### Pcp2-hShh mitigates the phenotype of disproportionately small cerebellum in Ts65Dn

Individuals with DS have a disproportionately small cerebellum [4], which is also present in various DS mouse models such as Ts65Dn, Ts1Cje, and TcMAC21 [42, 56]. To quantify the effects of increased Shh on cerebellar volume in Pcp2-hShh mice, a high-resolution 3D T2-weighted MRI was performed on the 14-month-old cerebellum-cohort (Fig. 7E). Two-way ANOVA analysis showed that the trisomy effect significantly reduced the cerebellum/brain ratio (*P* < 0.0001) and that cerebellar hShh overexpression significantly increased the cerebellum/brain ratio (*P* = 0.01). Tukey's post-hoc multiple comparisons showed that Pcp2-hShh mitigated the disproportionately small cerebellum in trisomic mice (*P* < 0.008 for Eu;TRE-hShh VS Ts65Dn;TRE-hShh, and *P* = 0.33 for Eu; TRE-hShh VS Ts65Dn;Pcp2-hShh, Fig. 7F). Relative to the average cerebellum/brain ratio of Eu;TRE-hShh (100%), it was 104.0% in Eu;Pcp2-hShh, 93.5% in Ts65Dn;TRE-hShh, and 96.9% in Ts65Dn;Pcp2-hShh (Fig. 7G).

## Discussion and conclusion

Shh protein plays an essential role in the development and maintenance of many organs/tissues. Previous conditional Shh knock-in mouse models including “(tetO)7CMV‐rShh” [54] and “CMV-lox-EGFP-STOP-lox-mShh” [80] were generated through random integration. The transgene status, such as the insertion location and copy number, is important, as random integration can cause endogenous gene mutations and the transgene copy number affects phenotypes. Here we used TARGATT site-specific transgenesis to generate a new, inducible Shh knock-in mouse “TRE-hShh”, which has one copy of TRE-hShh transgene in the neutral Rosa26 locus of chromosome 6. We show that the TRE-hShh mouse inducibly expresses the dually lipidated hShh-Np in the presence of tTA. The bidirectional TRE promoter provides a trackable Zsgreen1 marker in hShh-expressing cells. Using the human Shh transgene, we can distinguish the effects from endogenous or transgene expression and tell if they affect the expression of each other. Both human and mouse Shh-Np contain 174 amino acids, and the only amino acid difference between the two species is that S44 of hShh-Np is T44 in mShh-Np (Additional file 11: Figure S2C). Because serine could often be replaced by threonine without affecting protein function and vice versa, it is reasonable to expect very similar functions from hShh-Np and mShh-Np. With available tissue-specific rtTA or tTA driver lines, the TRE-hShh mouse could be used to study Shh function and its therapeutic potential in a wide range of tissues and cell types. Our findings and others [5, 87, 88] suggest that the tTA is likely more efficient than rtTA to drive TRE-transgene expression in brain.

ADHD affects 8.4% of all U.S. children 2–17 years of age [22], while the prevalence of ADHD could be as high as 40% in children with DS based on recent population-based studies [28, 57]. Children with ADHD reportedly have smaller and abnormally shaped basal ganglia, including caudate, putamen, and globus pallidus [38, 63]. Ts65Dn with locomotor hyperactivity shows alterations in morphology and plasticity of cholinergic interneurons in basal ganglia [26, 62]. Shh signaling is highly active in the developing ganglionic eminence (GE), where interneurons are produced and migrate to basal ganglia or cerebral cortex [24]. Here, we show that Camk2a-hShh but not Pcp2-hShh normalizes hyperactivity in Ts65Dn, consistent with the finding that Camk2a-tTA but not Pcp2-tTA induces TRE-transgene expression in basal ganglia (Additional file 11 (Figure S1G versus Figure S1H), and Fig. 3E versus Fig. 4C). Our findings support future studies on whether Shh response deficit exists in trisomic basal ganglia and whether it leads to hyperactivity in DS mouse models. To validate Shh therapeutic potential for ADHD, we need to elucidate the relationship between basal ganglia abnormalities, ADHD severities, and Shh response deficits.

Improving cognitive function is a major goal to enhance life opportunities for people with DS. Our previous SAG study shows that a single treatment of newborn Ts65Dn mice with SAG1.1 rescues phenotypes associated with hippocampal deficits (improved MWM performance and rescued hippocampal LTP) and cerebellar morphology in trisomic adults [23]. Here we extend those observations to show that Shh overexpression in forebrain rather than cerebellum significantly improves spatial learning and memory in 3-month-old Ts65Dn mice. Surprisingly, spatial learning was also improved in hShh-expressing euploid mice, which showed no enhancement in the previous SAG experiments. Moreover, aging Ts65Dn mice with forebrain Shh overexpression from the perinatal period do not show the same level of learning and memory loss seen in aging Ts65Dn. Although deficits in spatial memory of Ts65Dn are not completely rescued by forebrain Shh overexpression, the improvement is significant. Thus, we propose that Shh response deficits in forebrain (including hippocampus) rather than cerebellum are the dominant contributor to impaired spatial memory in Ts65Dn and that normalization of Shh signaling in forebrain structures during an appropriate developmental window has the therapeutic potential of mitigating intellectual disability in people with DS.

Gorlin syndrome (GS) is caused by loss-of-function mutations in *PTCH1*, resulting in upregulated Shh signaling, and 3–5% of people with GS develop childhood medulloblastoma [77]. Mice heterozygous for a null allele of *Ptch1* develop medulloblastoma beginning at an early age, and 14% are affected by 10 months of age [82]. Sporadic medulloblastoma sometimes presents with mutations in *SUFU* and *SMO* genes, as well [25]. However, all these mutations are in Shh-responding cells, causing constitutively active Shh signaling in the absence of Shh ligand. Up-regulation of Shh signaling by SAG shows therapeutic efficacy in various animal models, such as rats with spinal cord injury [7], mice with glucocorticoid-induced neonatal cerebellar injury [41], and Ts65Dn mice [23], and none of these studies show that SAG promotes tumor formation. Mouse Shh ligand overexpression in the mature exocrine pancreas by Ela-CreERT2;LSL-mShh or Ela-CreERT2;LSL-mShh;LSL-mSmo for up to 12 months does not induce neoplasia [31]. Here, neither hShh overexpression in forebrain by Camk2a-hShh for 600 days nor hShh overexpression in cerebellum by Pcp2-hShh for 14 months affects the survival rate in Eu or Ts65Dn. Thus, it is likely that in the absence of underlying mutations in Shh-responding cells, up-regulation of Shh signaling by Shh ligand or SAG is insufficient to induce carcinogenesis. Nevertheless, Shh signaling in various cancers merits attention in translating Shh research into clinical application.

We show that Gli1-LacZ labels neural precursors in neurogenic niches such as hippocampal DG and cerebellar oEGL and that Ts65Dn;Gli1-LacZ has reduced LacZ levels in both forebrain and cerebellum comparing with Eu;Gli1-LacZ at P6. As it can take over 2 weeks for LacZ protein to be fully degraded, the LacZ reduction in Ts65Dn;Gli1-LacZ is the accumulated readout from P6 to an earlier day. Interestingly, the 20% reduction of LacZ activity in cerebellum of P6 Ts65Dn;Gli1-LacZ is close to the 21% reduction in mitotic GCP of P0 Ts65Dn and the 28% reduction in total GCP of P6 Ts65Dn [66]. Our findings suggest that Ts65Dn mice have a neurogenic deficiency in the forebrain and cerebellum during the perinatal and early postnatal development that is associated with Shh signaling deficiency in these regions. It will be essential to explore whether people with DS and other neurological conditions have deficits in Shh-dependent neurogenesis and to further understand possible therapeutic windows, efficacy, toxicity, and formulation of Shh therapy.

## Supplementary Information

**Additional file 1. Video S1** Sagittal brain sections of 2-month-old Camk2a-hShh mice were immunostained with anti-Shh (red). Z-stack and tile scan images of the hippocampus region were converted into movies using Imaris. Two channels, DAPI (blue) and Zsgreen1 (green), were shown in (Video S1), and all three channels were shown in (Video S2).

**Additional file 2. Video S2** Sagittal brain sections of 2-month-old Camk2a-hShh mice were immunostained with anti-Shh (red). Z-stack and tile scan images of the hippocampus region were converted into movies using Imaris. Two channels, DAPI (blue) and Zsgreen1 (green), were shown in (Video S1), and all three channels were shown in (Video S2).

**Additional file 3. Video S3** Sagittal brain sections of 2-month-old Pcp2-hShh mice were immunostained with anti-Calbindin1. The cerebellum region was Z-stack and tile scan imaged with DAPI, Zsgreen, and RFP (anti-Calbindin1) confocal channels and converted into a movie.

**Additional file 4. Table S1** Key resources table.

**Additional file 5. Table S2** Experimental animal information.

**Additional file 6. Table S3** Oligonucleotides.

**Additional file 7. Table S4** Strategy to test Camk2a-rtTA;TRE-LacZ and Pcp2-rtTA;TRE-LacZ mice.

**Additional file 8. Table S5** Measurements from MRI of the cerebellum-cohort.

**Additional file 9. Table S6** Consolidated tables for all statistical analysis.

**Additional file 10. Table S7** RT-PCR of MEF cells with no plasmid transfection, CMV-rtTA and TRE-hShh transfection (-DOX), and CMV-rtTA and TRE-hShh transfection (+DOX).

**Additional file 11. Figure S1-7. **

## References

[1] Ahn S, Joyner AL (2004). Dynamic changes in the response of cells to positive hedgehog signaling during mouse limb patterning. Cell.

[2] Antonarakis SE, Skotko BG, Rafii MS, Strydom A, Pape SE, Bianchi DW, Sherman SL, Reeves RH (2020). Down syndrome. Nat Rev Dis Primers.

[3] Aoto K, Shikata Y, Imai H, Matsumaru D, Tokunaga T, Shioda S, Yamada G, Motoyama J (2009). Mouse Shh is required for prechordal plate maintenance during brain and craniofacial morphogenesis. Dev Biol.

[4] Aylward EH, Habbak R, Warren AC, Pulsifer MB, Barta PE, Jerram M, Pearlson GD (1997). Cerebellar volume in adults with Down syndrome. Arch Neurol.

[5] Badea TC, Hua ZL, Smallwood PM, Williams J, Rotolo T, Ye X, Nathans J (2009). New mouse lines for the analysis of neuronal morphology using CreER(T)/loxP-directed sparse labeling. PLoS ONE.

[6] Bai CB, Auerbach W, Lee JS, Stephen D, Joyner AL (2002). Gli2, but not Gli1, is required for initial Shh signaling and ectopic activation of the Shh pathway. Development.

[7] Bambakidis NC, Wang X, Lukas RJ, Spetzler RF, Sonntag VK, Preul MC (2010). Intravenous hedgehog agonist induces proliferation of neural and oligodendrocyte precursors in rodent spinal cord injury. Neurosurgery.

[8] Baxter LL, Moran TH, Richtsmeier JT, Troncoso J, Reeves RH (2000). Discovery and genetic localization of Down syndrome cerebellar phenotypes using the Ts65Dn mouse. Hum Mol Genet.

[9] Bayer KU, Lohler J, Schulman H, Harbers K (1999). Developmental expression of the CaM kinase II isoforms: ubiquitous gamma- and delta-CaM kinase II are the early isoforms and most abundant in the developing nervous system. Brain Res Mol Brain Res.

[10] Blassberg R, Macrae JI, Briscoe J, Jacob J (2016). Reduced cholesterol levels impair Smoothened activation in Smith-Lemli-Opitz syndrome. Hum Mol Genet.

[11] Brose RD, Lehrmann E, Zhang Y, Reeves RH, Smith KD, Mattson MP (2018). Hydroxyurea attenuates oxidative, metabolic, and excitotoxic stress in rat hippocampal neurons and improves spatial memory in a mouse model of Alzheimer's disease. Neurobiol Aging.

[12] Brose RD, Savonenko A, Devenney B, Smith KD, Reeves RH (2019). Hydroxyurea improves spatial memory and cognitive plasticity in mice and has a mild effect on these parameters in a down syndrome mouse model. Front Aging Neurosci.

[13] Cayuso J, Ulloa F, Cox B, Briscoe J, Marti E (2006). The Sonic hedgehog pathway independently controls the patterning, proliferation and survival of neuroepithelial cells by regulating Gli activity. Development.

[14] Chamberlain CE, Jeong J, Guo C, Allen BL, McMahon AP (2008). Notochord-derived Shh concentrates in close association with the apically positioned basal body in neural target cells and forms a dynamic gradient during neural patterning. Development.

[15] Chan JA, Balasubramanian S, Witt RM, Nazemi KJ, Choi Y, Pazyra-Murphy MF, Walsh CO, Thompson M, Segal RA (2009). Proteoglycan interactions with Sonic Hedgehog specify mitogenic responses. Nat Neurosci.

[16] Chen MH, Li YJ, Kawakami T, Xu SM, Chuang PT (2004). Palmitoylation is required for the production of a soluble multimeric Hedgehog protein complex and long-range signaling in vertebrates. Genes Dev.

[17] Chiang C, Litingtung Y, Lee E, Young KE, Corden JL, Westphal H, Beachy PA (1996). Cyclopia and defective axial patterning in mice lacking Sonic hedgehog gene function. Nature.

[18] Cooper JD, Salehi A, Delcroix JD, Howe CL, Belichenko PV, Chua-Couzens J, Kilbridge JF, Carlson EJ, Epstein CJ, Mobley WC (2001). Failed retrograde transport of NGF in a mouse model of Down's syndrome: reversal of cholinergic neurodegenerative phenotypes following NGF infusion. Proc Natl Acad Sci U S A.

[19] Cooper MK, Wassif CA, Krakowiak PA, Taipale J, Gong R, Kelley RI, Porter FD, Beachy PA (2003). A defective response to Hedgehog signaling in disorders of cholesterol biosynthesis. Nat Genet.

[20] Currier DG, Polk RC, Reeves RH, Dierssen MTFR (2012). Sonic hedgehog signaling as a therapeutic target for multiple features of Down syndrome. Progress in Brain Research.

[21] Dahmane N, Ruiz i Altaba A (1999). Sonic hedgehog regulates the growth and patterning of the cerebellum. Development.

[22] Danielson ML, Bitsko RH, Ghandour RM, Holbrook JR, Kogan MD, Blumberg SJ (2018). Prevalence of parent-reported ADHD diagnosis and associated treatment among U.S. children and adolescents, 2016. J Clin Child Adolesc Psychol.

[23] Das I, Park JM, Shin JH, Jeon SK, Lorenzi H, Linden DJ, Worley PF, Reeves RH (2013). Hedgehog agonist therapy corrects structural and cognitive deficits in a Down syndrome mouse model. Sci Transl Med.

[24] DeBoer EM, Anderson SA (2017). Fate determination of cerebral cortical GABAergic interneurons and their derivation from stem cells. Brain Res.

[25] Dellovade T, Romer JT, Curran T, Rubin LL (2006). The hedgehog pathway and neurological disorders. Annu Rev Neurosci.

[26] Di Filippo M, Tozzi A, Ghiglieri V, Picconi B, Costa C, Cipriani S, Tantucci M, Belcastro V, Calabresi P (2010). Impaired plasticity at specific subset of striatal synapses in the Ts65Dn mouse model of Down syndrome. Biol Psychiatry.

[27] Driscoll DA, Gross S (2009). Clinical practice. Prenatal screening for aneuploidy. N Engl J Med.

[28] Ekstein S, Glick B, Weill M, Kay B, Berger I (2011). Down syndrome and attention-deficit/hyperactivity disorder (ADHD). J Child Neurol.

[29] Escorihuela RM, Fernandez-Teruel A, Vallina IF, Baamonde C, Lumbreras MA, Dierssen M, Tobena A, Florez J (1995). A behavioral assessment of Ts65Dn mice: a putative Down syndrome model. Neurosci Lett.

[30] Faizi M, Bader PL, Tun C, Encarnacion A, Kleschevnikov A, Belichenko P, Saw N, Priestley M, Tsien RW, Mobley WC (2011). Comprehensive behavioral phenotyping of Ts65Dn mouse model of Down syndrome: activation of beta1-adrenergic receptor by xamoterol as a potential cognitive enhancer. Neurobiol Dis.

[31] Fendrich V, Oh E, Bang S, Karikari C, Ottenhof N, Bisht S, Lauth M, Brossart P, Katsanis N, Maitra A (2011). Ectopic overexpression of Sonic Hedgehog (Shh) induces stromal expansion and metaplasia in the adult murine pancreas. Neoplasia.

[32] Filges I, Rothlisberger B, Blattner A, Boesch N, Demougin P, Wenzel F, Huber AR, Heinimann K, Weber P, Miny P (2011). Deletion in Xp22.11: PTCHD1 is a candidate gene for X-linked intellectual disability with or without autism. Clin Genet.

[33] Fortress AM, Hamlett ED, Vazey EM, Aston-Jones G, Cass WA, Boger HA, Granholm ACE (2015). Designer receptors enhance memory in a mouse model of down syndrome. J Neurosci.

[34] Fujishima K, Horie R, Mochizuki A, Kengaku M (2012). Principles of branch dynamics governing shape characteristics of cerebellar Purkinje cell dendrites. Development.

[35] Furth PA, St Onge L, Boger H, Gruss P, Gossen M, Kistner A, Bujard H, Hennighausen L (1994). Temporal control of gene expression in transgenic mice by a tetracycline-responsive promoter. Proc Natl Acad Sci U S A.

[36] Gonzalez-Reyes LE, Verbitsky M, Blesa J, Jackson-Lewis V, Paredes D, Tillack K, Phani S, Kramer ER, Przedborski S, Kottmann AH (2012). Sonic hedgehog maintains cellular and neurochemical homeostasis in the adult nigrostriatal circuit. Neuron.

[37] Goodrich LV, Johnson RL, Milenkovic L, McMahon JA, Scott MP (1996). Conservation of the hedgehog/patched signaling pathway from flies to mice: induction of a mouse patched gene by Hedgehog. Genes Dev.

[38] Halperin JM, Schulz KP (2006). Revisiting the role of the prefrontal cortex in the pathophysiology of attention-deficit/hyperactivity disorder. Psychol Bull.

[39] Harfe BD, Scherz PJ, Nissim S, Tian H, McMahon AP, Tabin CJ (2004). Evidence for an expansion-based temporal Shh gradient in specifying vertebrate digit identities. Cell.

[40] He P, Staufenbiel M, Li R, Shen Y (2014). Deficiency of patched 1-induced Gli1 signal transduction results in astrogenesis in Swedish mutated APP transgenic mice. Hum Mol Genet.

[41] Heine VM, Griveau A, Chapin C, Ballard PL, Chen JK, Rowitch DH (2011). A small-molecule smoothened agonist prevents glucocorticoid-induced neonatal cerebellar injury. Sci Transl Med.

[42] Kazuki Y, Gao FJ, Li Y, Moyer AJ, Devenney B, Hiramatsu K, Miyagawa-Tomita S, Abe S, Kazuki K, Kajitani N (2020). A non-mosaic transchromosomic mouse model of down syndrome carrying the long arm of human. Chromosome.

[43] Koirala S, Corfas G (2010). Identification of novel glial genes by single-cell transcriptional profiling of Bergmann glial cells from mouse cerebellum. PLoS ONE.

[44] Korenberg JR, Chen XN, Schipper R, Sun Z, Gonsky R, Gerwehr S, Carpenter N, Daumer C, Dignan P, Disteche C (1994). Down syndrome phenotypes: the consequences of chromosomal imbalance. Proc Natl Acad Sci U S A.

[45] Krestel HE, Mayford M, Seeburg PH, Sprengel R (2001). A GFP-equipped bidirectional expression module well suited for monitoring tetracycline-regulated gene expression in mouse. Nucleic Acids Res.

[46] Kruszka P, Muenke M (2018). Syndromes associated with holoprosencephaly. Am J Med Genet C Semin Med Genet.

[47] Lewis PM, Dunn MP, McMahon JA, Logan M, Martin JF, St-Jacques B, McMahon AP (2001). Cholesterol modification of sonic hedgehog is required for long-range signaling activity and effective modulation of signaling by Ptc1. Cell.

[48] Lewis PM, Gritli-Linde A, Smeyne R, Kottmann A, McMahon AP (2004). Sonic hedgehog signaling is required for expansion of granule neuron precursors and patterning of the mouse cerebellum. Dev Biol.

[49] Li Y, Zhang H, Litingtung Y, Chiang C (2006). Cholesterol modification restricts the spread of Shh gradient in the limb bud. Proc Natl Acad Sci U S A.

[50] Machold R, Hayashi S, Rutlin M, Muzumdar MD, Nery S, Corbin JG, Gritli-Linde A, Dellovade T, Porter JA, Rubin LL (2003). Sonic hedgehog is required for progenitor cell maintenance in telencephalic stem cell niches. Neuron.

[51] Mansuy IM, Winder DG, Moallem TM, Osman M, Mayford M, Hawkins RD, Kandel ER (1998). Inducible and reversible gene expression with the rtTA system for the study of memory. Neuron.

[52] Martinez S, Andreu A, Mecklenburg N, Echevarria D (2013). Cellular and molecular basis of cerebellar development. Front Neuroanat.

[53] Mayford M, Bach ME, Huang YY, Wang L, Hawkins RD, Kandel ER (1996). Control of memory formation through regulated expression of a CaMKII transgene. Science.

[54] Miller LA, Wert SE, Clark JC, Xu Y, Perl AK, Whitsett JA (2004). Role of Sonic hedgehog in patterning of tracheal-bronchial cartilage and the peripheral lung. Dev Dyn.

[55] Niewiadomski P, Niedziolka SM, Markiewicz L, Uspienski T, Baran B, Chojnowska K (2019). Gli Proteins: Regulation in Development and Cancer. Cells.

[56] Olson LE, Roper RJ, Baxter LL, Carlson EJ, Epstein CJ, Reeves RH (2004). Down syndrome mouse models Ts65Dn, Ts1Cje, and Ms1Cje/Ts65Dn exhibit variable severity of cerebellar phenotypes. Dev Dyn.

[57] Oxelgren UW, Myrelid A, Anneren G, Ekstam B, Goransson C, Holmbom A, Isaksson A, Aberg M, Gustafsson J, Fernell E (2017). Prevalence of autism and attention-deficit-hyperactivity disorder in Down syndrome: a population-based study. Dev Med Child Neurol.

[58] Palma V, Lim DA, Dahmane N, Sanchez P, Brionne TC, Herzberg CD, Gitton Y, Carleton A, Alvarez-Buylla A, Ruiz i Altaba A,  (2005). Sonic hedgehog controls stem cell behavior in the postnatal and adult brain. Development.

[59] Petrova R, Joyner AL (2014). Roles for Hedgehog signaling in adult organ homeostasis and repair. Development.

[60] Placzek M (1995). The role of the notochord and floor plate in inductive interactions. Curr Opin Genet Dev.

[61] Porter JA, Young KE, Beachy PA (1996). Cholesterol modification of hedgehog signaling proteins in animal development. Science.

[62] Powers BE, Velazquez R, Kelley CM, Ash JA, Strawderman MS, Alldred MJ, Ginsberg SD, Mufson EJ, Strupp BJ (2016). Attentional function and basal forebrain cholinergic neuron morphology during aging in the Ts65Dn mouse model of Down syndrome. Brain Struct Funct.

[63] Qiu A, Crocetti D, Adler M, Mahone EM, Denckla MB, Miller MI, Mostofsky SH (2009). Basal ganglia volume and shape in children with attention deficit hyperactivity disorder. Am J Psychiatry.

[64] Rahimi-Balaei M, Bergen H, Kong J, Marzban H (2018). Neuronal Migration During Development of the Cerebellum. Front Cell Neurosci.

[65] Roessler E, Belloni E, Gaudenz K, Jay P, Berta P, Scherer SW, Tsui LC, Muenke M (1996). Mutations in the human Sonic Hedgehog gene cause holoprosencephaly. Nat Genet.

[66] Roper RJ, Baxter LL, Saran NG, Klinedinst DK, Beachy PA, Reeves RH (2006). Defective cerebellar response to mitogenic Hedgehog signaling in Down's syndrome mice. P Natl Acad Sci USA.

[67] Roper RJ, VanHorn JF, Cain CC, Reeves RH (2009). A neural crest deficit in Down syndrome mice is associated with deficient mitotic response to Sonic hedgehog. Mech Dev.

[68] Sasai N, Toriyama M, Kondo T (2019). Hedgehog Signal and Genetic Disorders. Front Genet.

[69] Sergaki MC, Ibanez CF (2017). GFRalpha1 regulates purkinje cell migration by counteracting NCAM function. Cell Rep.

[70] Shao S, Wang GL, Raymond C, Deng XH, Zhu XL, Wang D, Hong LP (2017). Activation of Sonic hedgehog signal by Purmorphamine, in a mouse model of Parkinson's disease, protects dopaminergic neurons and attenuates inflammatory response by mediating PI3K/AKt signaling pathway. Mol Med Rep.

[71] Solomon BD, Rosenbaum KN, Meck JM, Muenke M (2010). Holoprosencephaly due to numeric chromosome abnormalities. Am J Med Genet C.

[72] St-Jacques B, Dassule HR, Karavanova I, Botchkarev VA, Li J, Danielian PS, McMahon JA, Lewis PM, Paus R, McMahon AP (1998). Sonic hedgehog signaling is essential for hair development. Curr Biol.

[73] Tasic B, Hippenmeyer S, Wang C, Gamboa M, Zong H, Chen-Tsai Y, Luo L (2011). Site-specific integrase-mediated transgenesis in mice via pronuclear injection. Proc Natl Acad Sci U S A.

[74] Traiffort E, Angot E, Ruat M (2010). Sonic Hedgehog signaling in the mammalian brain. J Neurochem.

[75] Traiffort E, Charytoniuk D, Watroba L, Faure H, Sales N, Ruat M (1999). Discrete localizations of hedgehog signalling components in the developing and adult rat nervous system. Eur J Neurosci.

[76] Tsien JZ, Chen DF, Gerber D, Tom C, Mercer EH, Anderson DJ, Mayford M, Kandel ER, Tonegawa S (1996). Subregion- and cell type-restricted gene knockout in mouse brain. Cell.

[77] Vorechovsky I, Tingby O, Hartman M, Stromberg B, Nister M, Collins VP, Toftgard R (1997). Somatic mutations in the human homologue of Drosophila patched in primitive neuroectodermal tumours. Oncogene.

[78] Vorobyeva AG, Saunders AJ (2018). Amyloid-beta interrupts canonical Sonic hedgehog signaling by distorting primary cilia structure. Cilia.

[79] Wallace VA (1999). Purkinje-cell-derived Sonic hedgehog regulates granule neuron precursor cell proliferation in the developing mouse cerebellum. Curr Biol.

[80] Wang DH, Clemons NJ, Miyashita T, Dupuy AJ, Zhang W, Szczepny A, Corcoran-Schwartz IM, Wilburn DL, Montgomery EA, Wang JS (2010). Aberrant epithelial-mesenchymal Hedgehog signaling characterizes Barrett's metaplasia. Gastroenterology.

[81] Wang M, Marco P, Capra V, Kibar Z (2019). Update on the role of the non-canonical Wnt/planar cell polarity pathway in neural tube defects. Cells.

[82] Wetmore C, Eberhart DE, Curran T (2000). The normal patched allele is expressed in medulloblastomas from mice with heterozygous germ-line mutation of patched. Cancer Res.

[83] Yabut OR, Pleasure SJ (2018). Sonic hedgehog signaling rises to the surface: emerging roles in neocortical development. Brain Plast.

[84] Yao PJ, Petralia RS, Mattson MP (2016). Sonic hedgehog signaling and hippocampal neuroplasticity. Trends Neurosci.

[85] Ybot-Gonzalez P, Gaston-Massuet C, Girdler G, Klingensmith J, Arkell R, Greene ND, Copp AJ (2007). Neural plate morphogenesis during mouse neurulation is regulated by antagonism of Bmp signalling. Development.

[86] Zeng X, Goetz JA, Suber LM, Scott WJ, Schreiner CM, Robbins DJ (2001). A freely diffusible form of Sonic hedgehog mediates long-range signalling. Nature.

[87] Zhu P, Aller MI, Baron U, Cambridge S, Bausen M, Herb J, Sawinski J, Cetin A, Osten P, Nelson ML (2007). Silencing and un-silencing of tetracycline-controlled genes in neurons. PLoS ONE.

[88] Zou Y, Chen CH, Fike JR, Huang TT (2009). A new mouse model for temporal- and tissue-specific control of extracellular superoxide dismutase. Genesis.

[89] Zu T, Duvick LA, Kaytor MD, Berlinger MS, Zoghbi HY, Clark HB, Orr HT (2004). Recovery from polyglutamine-induced neurodegeneration in conditional SCA1 transgenic mice. J Neurosci.

