# Supplementary material for: Forebrain Shh overexpression improves cognitive function and locomotor hyperactivity in an aneuploid mouse model of Down syndrome and its euploid littermates
Source: Acta Neuropathol Commun. 2021 Aug 16;9:137. doi: 10.1186/s40478-021-01237-z (PMC8365939; doi:10.1186/s40478-021-01237-z)
Supplement: Supplementary file 11 — Additional file 11. Figure S1-7. [file 40478_2021_1237_MOESM11_ESM.docx]

**Additional File 11**

**Figures S1-7**

- **Figure S1.** Animal models creation and verification, related to Figure 2
- **Figure S2.** Camk2a-tTA induces TRE-hShh to express hShh in mice, related to Figure 3
- **Figure S3.** FACS of cerebral cortex and Hippocampus from of Pcp2-hShh without Dox treatment, related to Figure 4
- **Figure S4.** The patterns of transgene inheritance and expression, related to Figure 5
- **Figure S5.** Visual discrimination task, related to Figure 6
- **Figure S6.** Supplements for MWM, related to Figure 6
- **Figure S7.** Supplements for RRWM, related to Figure 7


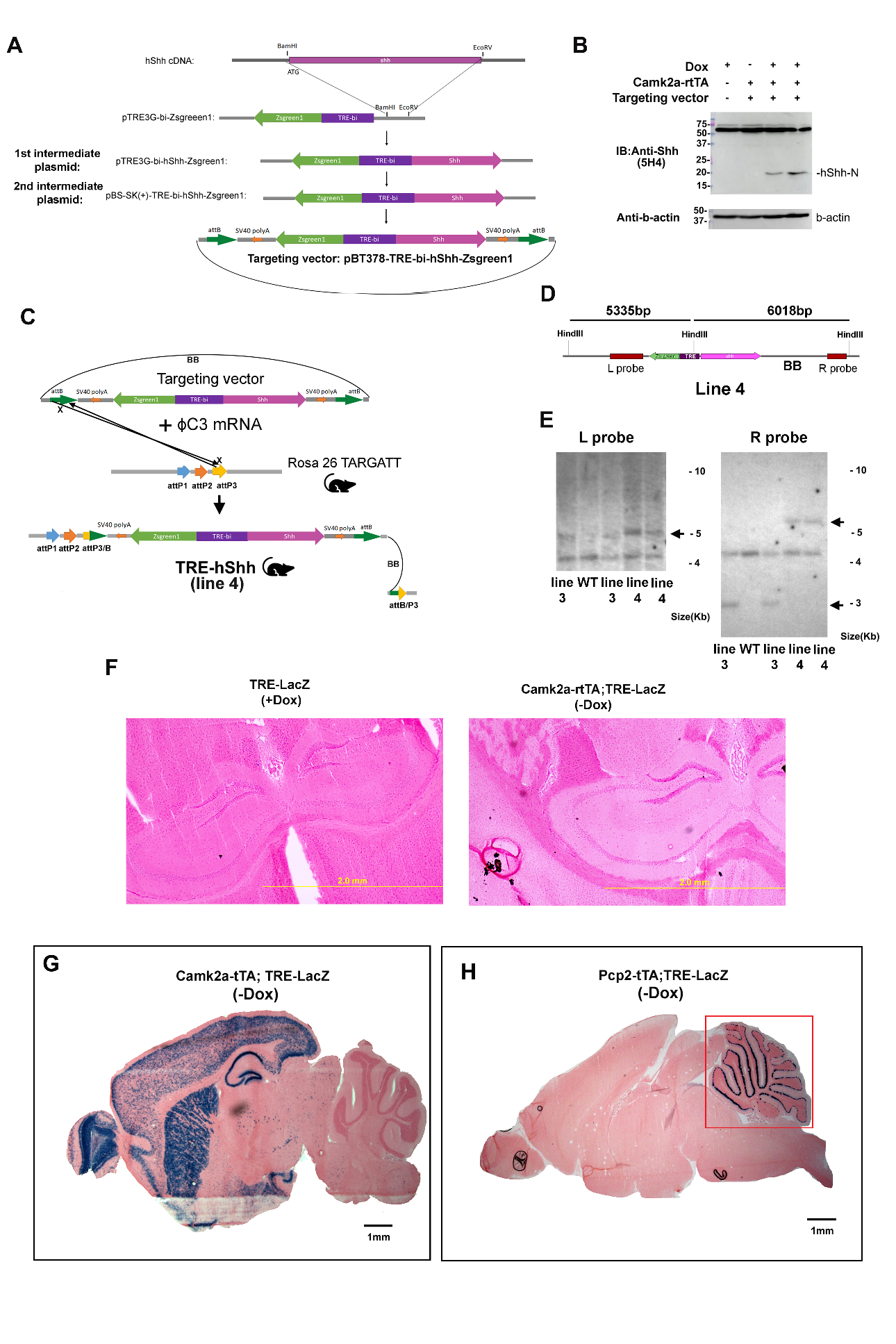


**Figure S1. Animal models creation and verification, related to Figure 2**

1. Scheme illustrating the generation of the targeting vector, TRE-bi-hShh-Zsgreen1. For the first intermediate plasmid generation, “pTRE3G-bi-hShh-Zsgreen1”, full-length hShh cDNA was amplified from HsCD00082632 (DNASU) with primers of Shh-halfRV and Shh-Bam2, which was digested with BamHI and then ligated with pTRE3G-bi-ZsGreen1 after BamHI/EcoRV digestion. To generate the intermediate plasmid, “pBS-SK(+)-TRE-bi-hShh-Zsgreen1”*,* pTRE3G-bi-hShh-Zsgreen1 was digested with PciI and filled-in using Klenow followed by EcoRI digestion to create the TRE-hShh cassette, which was ligated with pBS-SK(+) after SmaI/EcoRI digestion to create pBS-SK(+)-TRE-hShh, and Zsgreen1/polyA was amplified from pTRE3G-bi-ZsGreen1 with primers of ClaI-F_Zs2 and RI-R_Zs and ligated with pBS-SK(+)-TRE-hShh after ClaI/EcoRI digestion. To generate the Targeting vector, pBS-SK(+)-TRE-bi-hShh-Zsgreen1 was digested with ClaI and Not I to get the fragment “TRE-bi-hShh-Zsgreen1 with polyAs”, which was ligated with pBT378 after ClaI/Not I digestion.
2. Western blot of MEFs co-transfected with CMV-rtTA and targeting vectors and treated with or without Dox using anti-N-terminus Shh antibodies (5H4).
3. Scheme illustrating that TRE-hShh line 4 had the whole plasmid (with BB) inserted at attP3/attP3.
4. Model of predicting southern blot of TRE-hShh (line4). HindIII digestion produces two fragments. Because of containing two extra attP sites, the left fragment of line 4 was 5335bp, slightly larger than the 5193bp-fragment of line 2 and 3. Because of BB, the right fragment was 6018bp, which was significantly larger than 2857bp fragments of line 2 and 3.
5. Southern blots of WT and TRE-hShh (line 3 and 4) mice using radiolabeled L and R probes.
6. Representative images of X-gal stained coronal brain sections from P30 TRE-LacZ mice with Dox treatment from conception (625 mg/Kg food pellets plus 3.5 mg/ml in drinking water) and Camk2a-rtTA;TRE-LacZ without Dox. Hippocampus was shown.
7. Representative images of X-gal stained sagittal brain sections from P30 Camk2a-tTA;TRE-LacZ mice without Dox treatment.
8. Representative images of X-gal stained sagittal brain sections from P14 Pcp2-tTA;TRE-LacZ mice without Dox treatment.


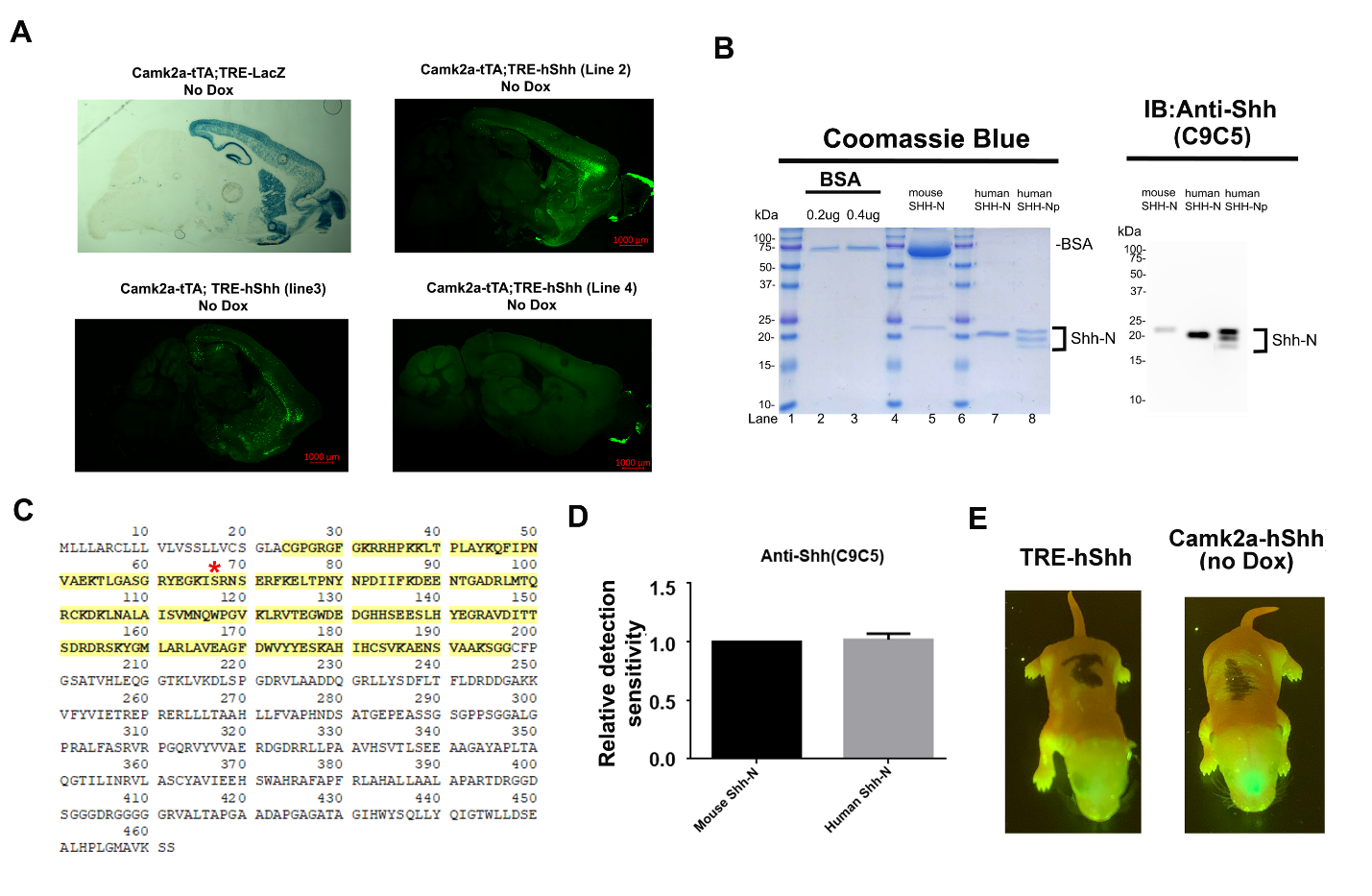


**Figure S2. Camk2a-tTA induces TRE-hShh to express hShh in mice, related to Figure 3**

1. Sagittal brain sections of Camk2a-tTA;TRE-hShh (Camk2a-hShh), from lines 2, 3, and 4 mice at 2-month-old that were imaged in Zsgreen1 channel, and the tile scan confocal images were shown. X-gal staining of sagittal brain sections of Camk2a-tTA;TRE-LacZ mice were used as the comparison. Scale bar 1000 um.
2. The purified recombinant mShh-N (6-His-tagged, R&D 461SH), hShh-N (IVI at N-terminus, PeproTECH 100-45), and hShh-Np (R&D 8908-SH/CF) were shown in Coomassie blue staining (left) and Western blot of anti-Shh (right). The total loaded protein in Coomassie blue staining was 50 times as much as that in Western blot.
3. Full-length hShh protein sequence. The C24-G197 (yellow highlighted) is the Shh-N with an MW of 19.56 kDa. Palmitoylation at C24 adds ~238 Da, and cholesterol addition at G197 is ~369 Da. Red * points to the only amino acid difference between hShh-N and mShh-N.
4. Quantitative analysis of C9C5’s relative detection sensitivity of mShh-N to hShh-N. Purified recombinant protein of mShh-N (6-His-tagged, R&D 461SH) and hShh-N (IVI at N-terminus, PeproTECH 100-45) were compared by Western blot.
5. P1 TRE-hShh and Camk2a-hShh pups without Dox treatment were visualized by GFP flashlight.


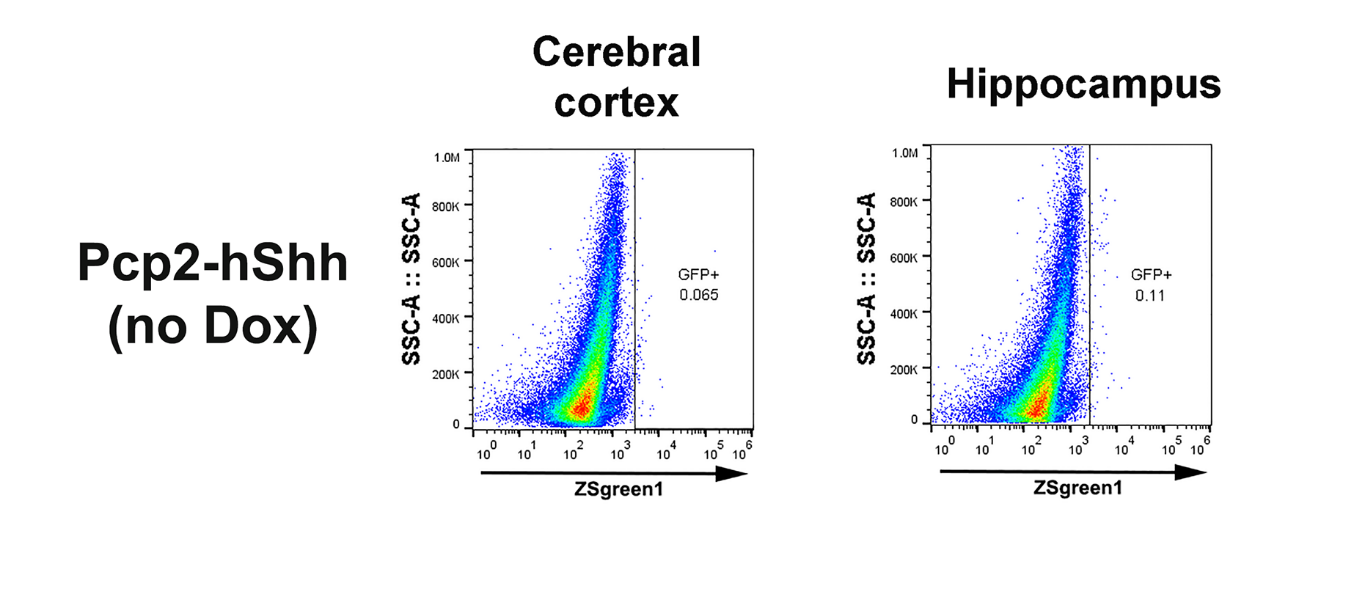


**Figure S3. FACS of cerebral cortex and Hippocampus from of Pcp2-hShh without Dox treatment, related to Figure 4**


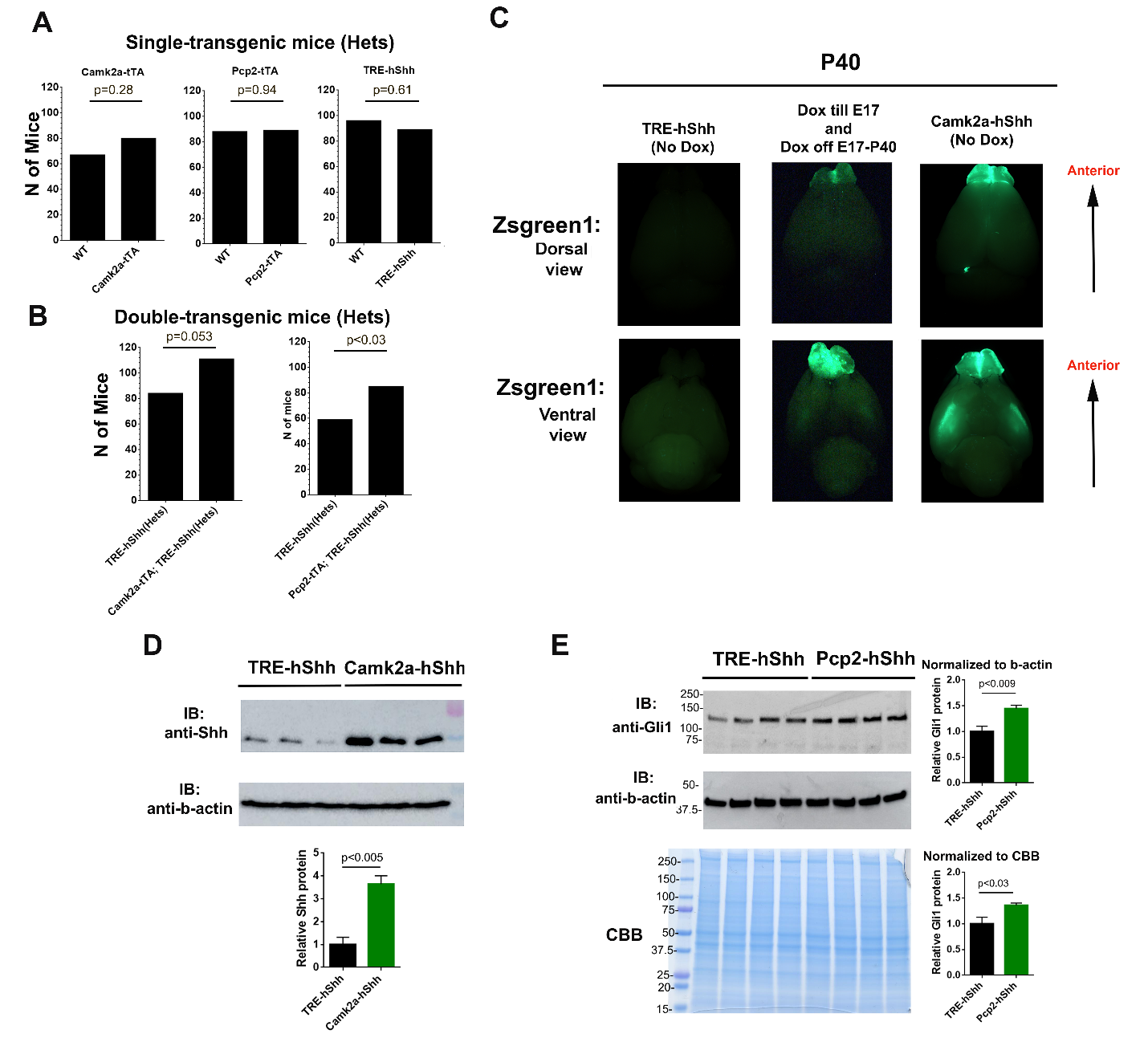


**Figure S4. The patterns of transgene inheritance and expression, related to Figure 5**

1. The offspring ratio of single transgenic mice. The breeding records of Camk2a-tTA, Pcp2-tTA, and TRE-hShh single transgenic mice were analyzed at weaning age. Data were analyzed by Binomial tests.
2. The offspring ratio of double transgenic mice, Camk2a-tTA;TRE-hShh and Pcp2-tTA-hShh, to TRE-hShh was compared. Data were analyzed by Binomial tests.
3. P40 mouse brains of TRE-hShh (left), Camk2a-hShh with “E0-E17” Dox treatment (middle), and Camk2a-hShh without Dox treatment (right) were visualized in GFP-channel.
4. Western blot of cortex from TRE-hShh and Camk2-hShh at 3-month-old. The quantification of Shh density was analyzed by unpaired t-tests (n=3).
5. Western blot of cerebellum from TRE-hShh and Pcp2-hShh mice. The quantification of Gli1 density was analyzed by unpaired t-tests (n=4).


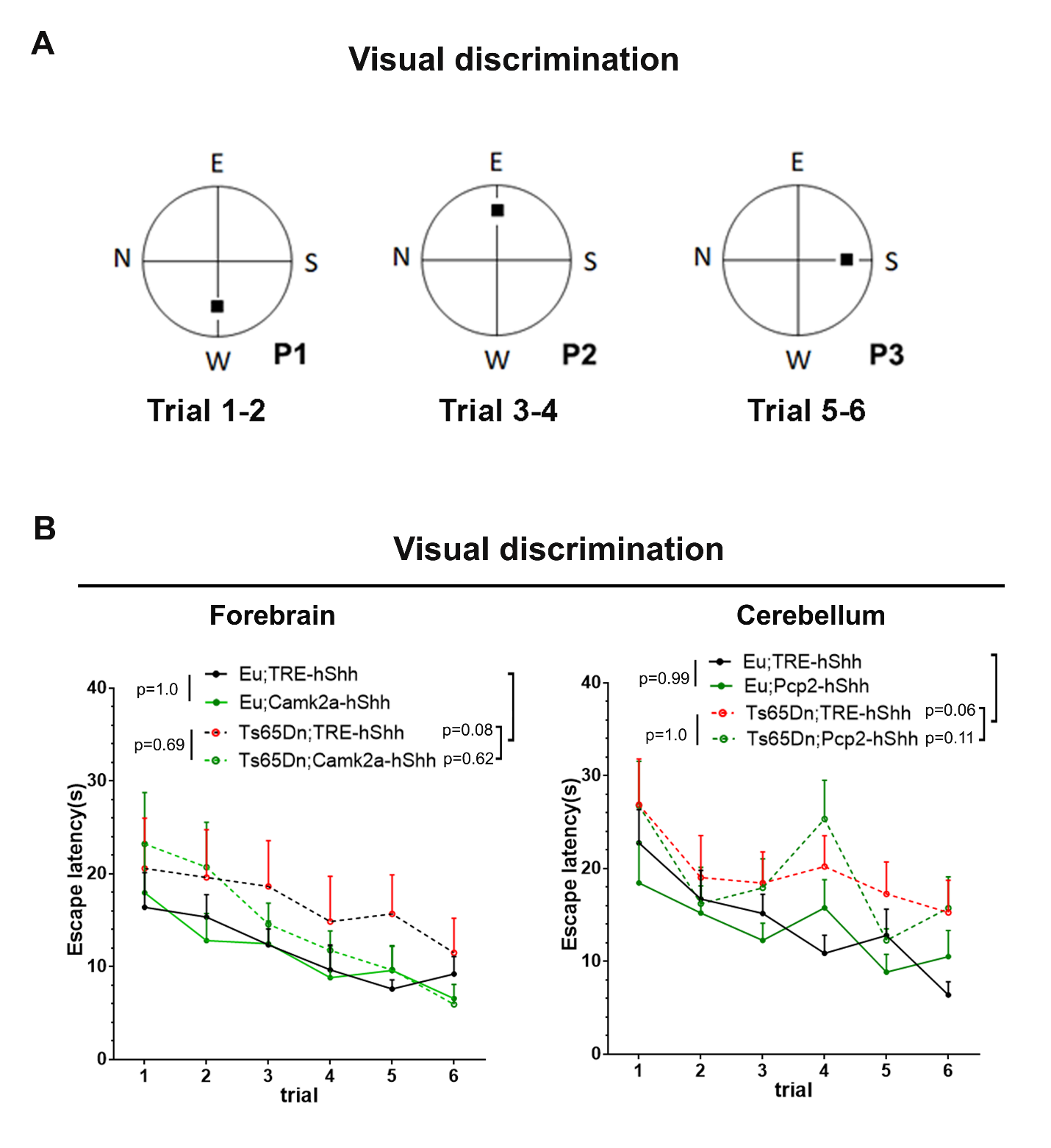


**Figure S5. Visual discrimination task, related to Figure 6**

1. Scheme illustrating six-trial visual discrimination task.
2. Visual discrimination task results of the forebrain-cohort (left) and the cerebellum-cohort (right). Data were represented as mean ± SEM and analyzed by two-way RM AVOVA and Tukey's multiple comparisons test.


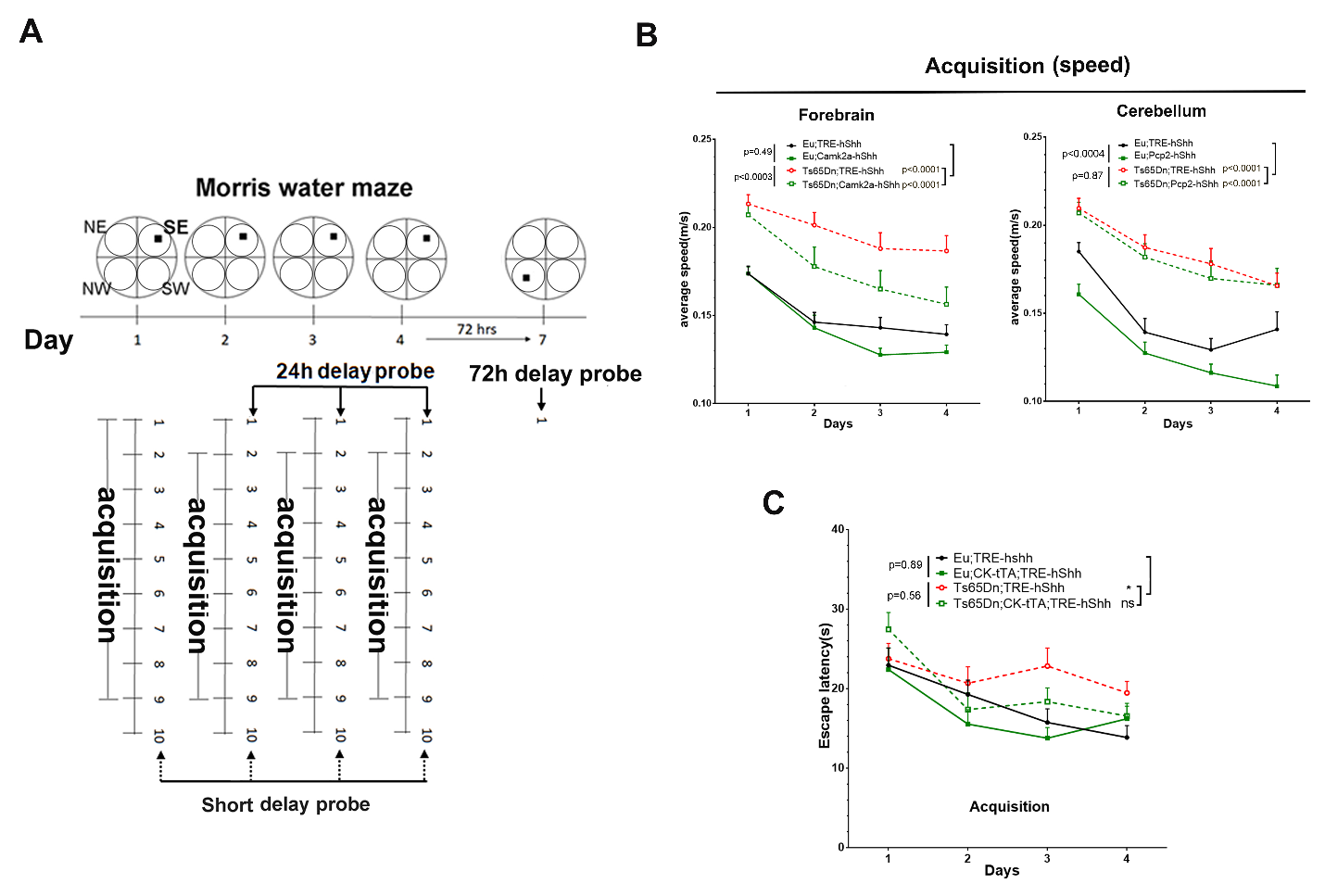


**Figure S6. Supplements for MWM, related to Figure 6**

1. Scheme illustrating classic MWM design.
2. Swimming speed of acquisition trials (average speed of 8 trials per day).
3. Escape latency in acquisition trials of MWM (average latency of 8 trials each day).

Data are analyzed by two-way ANOVA and Tukey's multiple comparisons test and expressed as mean ± SEM.


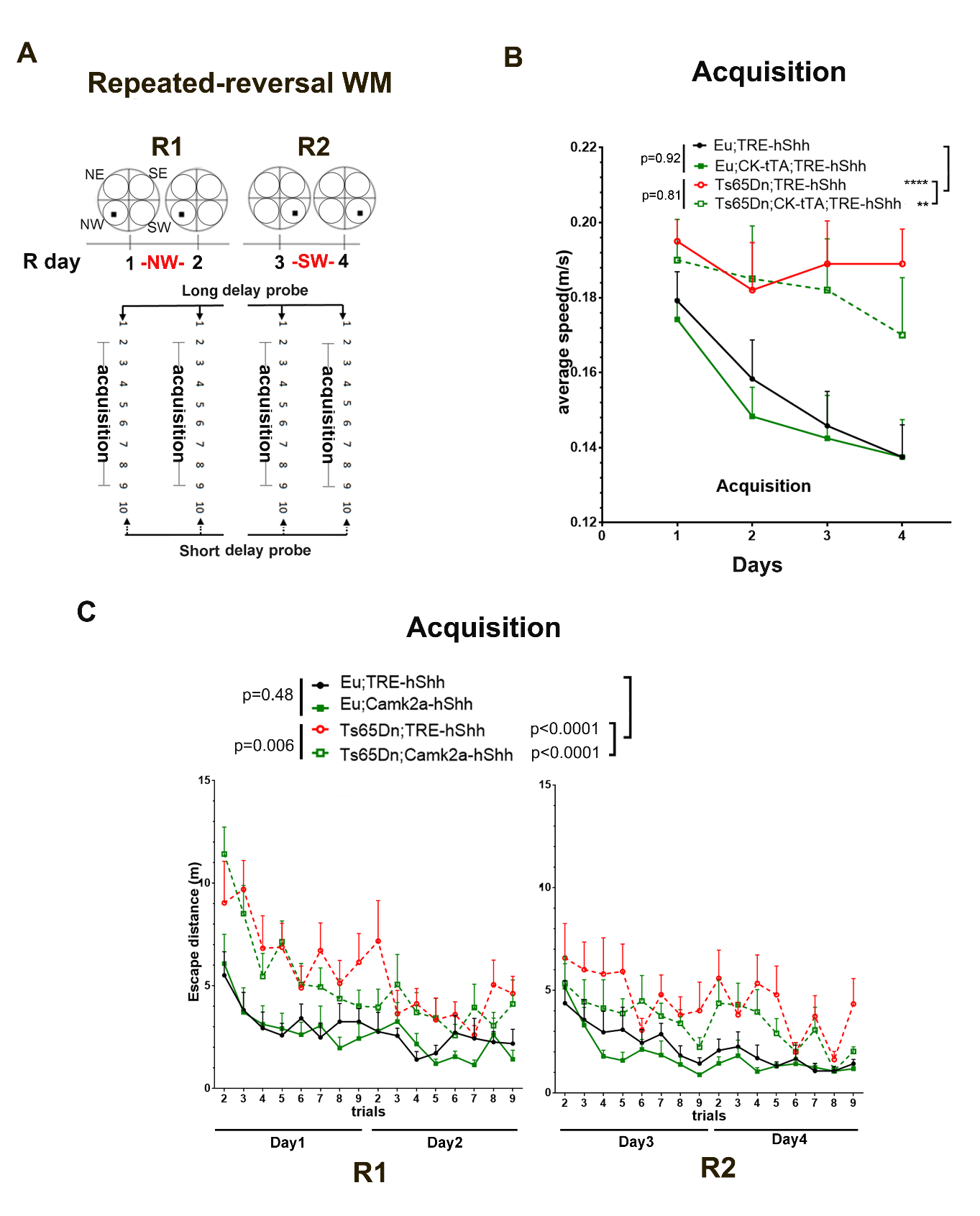


**Figure S7. Supplements for RRWM, related to Figure 7**

1. Scheme illustrating RRWM.
2. Swimming speed in acquisition trials of RRWM.
3. Escape distance in acquisition trials of RRWM.

Data are analyzed by two-way ANOVA and Tukey's multiple comparisons test and expressed as mean ± SEM.
